# The Systemic Immune State of Super-shedder Mice Is Characterized by a Unique Neutrophil-dependent Blunting of T_H_1 Responses

**DOI:** 10.1371/journal.ppat.1003408

**Published:** 2013-06-06

**Authors:** Smita Gopinath, Andrew Hotson, Jennifer Johns, Garry Nolan, Denise Monack

**Affiliations:** 1 Department of Microbiology and Immunology, Stanford University School of Medicine, Stanford, California, United States of America; 2 Department of Microbiology and Immunology, The Baxter Laboratory of Genetic Pharmacology, Stanford University School of Medicine, Stanford, California, United States of America; 3 Department of Comparative Medicine, Stanford University School of Medicine, Stanford, California, United States of America; Yale University School of Medicine, United States of America

## Abstract

Host-to-host transmission of a pathogen ensures its successful propagation and maintenance within a host population. A striking feature of disease transmission is the heterogeneity in host infectiousness. It has been proposed that within a host population, 20% of the infected hosts, termed super-shedders, are responsible for 80% of disease transmission. However, very little is known about the immune state of these super-shedders. In this study, we used the model organism *Salmonella enterica* serovar Typhimurium, an important cause of disease in humans and animal hosts, to study the immune state of super-shedders. Compared to moderate shedders, super-shedder mice had an active inflammatory response in both the gastrointestinal tract and the spleen but a dampened T_H_1 response specific to the secondary lymphoid organs. Spleens from super-shedder mice had higher numbers of neutrophils, and a dampened T cell response, characterized by higher levels of regulatory T cells (T_regs_), fewer T-bet^+^ (T_H_1) T cells as well as blunted cytokine responsiveness. Administration of the cytokine granulocyte colony stimulating factor (G-CSF) and subsequent neutrophilia was sufficient to induce the super-shedder immune phenotype in moderate-shedder mice. Similar to super-shedders, these G-CSF-treated moderate-shedders had a dampened T_H_1 response with fewer T-bet^+^ T cells and a loss of cytokine responsiveness. Additionally, G-CSF treatment inhibited IL-2-mediated T_H_1 expansion. Finally, depletion of neutrophils led to an increase in the number of T-bet^+^ T_H_1 cells and restored their ability to respond to IL-2. Taken together, we demonstrate a novel role for neutrophils in blunting IL-2-mediated proliferation of the T_H_1 immune response in the spleens of mice that are colonized by high levels of *S.* Typhimurium in the gastrointestinal tract.

## Author Summary

Bacteria belonging to the genus *Salmonella* are capable of causing long-term chronic systemic infections in specific hosts where they are shed in the feces. These persistently infected individuals include typhoid carriers and they serve as a reservoir for disease transmission. Despite the importance of *Salmonella* as a human pathogen, relatively little is known about the host immune response to persistent bacterial infections in the context of transmission. We had shown previously in a mouse model of *Salmonella* infection that mice shedding high levels of *Salmonella* (>10^8^ bacteria per gram of feces), known as super-shedders, transmit disease to naïve mice. We show here that these super-shedder mice have a unique immune state compared to mice that have lower levels of *Salmonella* in their gut. The super-shedder immune state is characterized by an active inflammatory immune response with elevated serum IL-6 and high levels of neutrophils in both the gastrointestinal tract and the systemic sites but a dampened adaptive CD4 T helper type1 (T_H_1) cell response specific to the spleen. Importantly, we show that the blunted adaptive response, as characterized by reduced T_H_1 cell frequencies and ability to respond to IL-2 and IL-6, is intimately linked to the levels of neutrophils present in the spleen. We go on to show the functional consequences of dampened cytokine responsiveness, as T_H_1 cells from moderate-shedders are unable to undergo IL-2-mediated expansion when neutrophilia is induced. Additionally, we show that neutrophil control of IL-2 mediated expansion of T_H_1 cells is independent of infection. In summary, we describe an immune phenotype associated with transmission of a pathogen and a single immune cell type, neutrophils, which control specific aspects of the super-shedder immune state.

## Introduction

Host-adapted pathogens depend on their host for transmission and dissemination within a population. Recent epidemiological studies have uncovered heterogeneities in infection wherein a minority of the infected individuals (20%) are responsible for the majority of the infections (80%), described as the 80/20 rule [1]. In the case of pathogens transmitted via the fecal-oral route, these individuals are the ones that shed the highest numbers of bacteria. Recent studies on the transmission of *Escherichia coli* O157 within cattle herds demonstrated that over 95% of the infections were caused by between 8–10% of the most infectious individuals, or super-shedders [2]–[4]. Identification of these individuals is required for control of the infection [1], [5], [6]. However, little is known about what distinguishes them from other infected hosts.

*Salmonella enterica* serovar Typhi, the causative agent of typhoid fever in humans, is a human-adapted pathogen and establishes a persistent long-term infection in about 1–6% of the infected hosts [7], [8]. These individuals are known as typhoid carriers and periodically excrete large amounts of the bacilli in their feces, thereby offering both a reservoir for the pathogen and the opportunity of transmission to new hosts. However, they do not display any of the clinical signs characteristically associated with typhoid fever [7], [9]. While individuals with acute infections can transmit the pathogen for brief periods of time, for the purposes of this study, we will focus on transmission from persistently infected hosts who play a far larger role in transmission of host-adapted pathogens [1], [2], [4], [10].

Host immune responses to persistent microbial infections must balance between control of the pathogen and minimizing inflammatory damage to the host [11]. To this end, chronic viral infections often result in a contraction of the adaptive immune response, an example of which would be T cell exhaustion [12], [13]. An ineffective CD4 T cell response, characterized by anergy or apoptosis, has also been observed in persistent bacterial infections such as with *Helicobacter pylori*, *Staphylococcus aureus* and *Salmonella enterica* serovar Typhimurium [14]–[16]. Intriguingly, regulatory T cells have also been shown to lose their suppressive ability during the later stages of persistent *S*. Typhimurium infection [17].

Characterizing the host immune response in individuals that transmit disease is important for two reasons. First, understanding the mechanistic differences in this subset of hosts might explain the heterogeneity of infectiousness observed in relatively homogenous populations, such as with inbred herds of cattle. Second, such studies could lead to the development of biomarkers that are unique to the identification of individuals with the highest risk of transmitting the pathogen within a population.

Modeling the immune state using laboratory animals allows us to dissect the mechanisms behind host-pathogen interactions that lead to transmission. Our lab has established a mouse model of persistent *S.* Typhimurium infection wherein 30% of the infected mice, termed super-shedders, shed >10^8^ *Salmonella* and rapidly transmit disease to naïve cage mates [18]. This variation in infectiousness is observed in inbred strains amongst cage mates and siblings, implying predisposition to super-shedder status might not be heritable. Super-shedder mice also develop colitis, displaying moderate to severe inflammation in the colon and ceca. Surprisingly, super-shedder mice do not display outward signs of illness such as ruffled fur, fever or malaise [18] suggesting that they can tolerate, and perhaps control the inflammation. How, then, does the interplay between pathogen and the murine immune system evolve to allow such high levels of gastrointestinal *Salmonella* in some mice, but not others?

The host immune response to *Salmonella* infections has been characterized primarily in mice that have increased susceptibility to intracellular pathogens due to the presence of a mutated Nramp1 gene [19]–[21]. In susceptible mice, wild type *S.* Typhimurium infection results in death within one week of infection. In contrast, these susceptible mice strains survive infection with attenuated *S.* Typhimurium strains (e.g, AroA^−^ AroD^−^ mutant). In this model the adaptive immune response to persistent *Salmonella* infection was found to be T_H_1 biased and was dependent upon expression of transcription factor T-bet [22].

In this study, we used a mouse strain (129x1/SvJ) that carries functional Nramp1 and a non-attenuated Salmonella strain to more closely mimic natural infection [23]. This persistent infection model was used to investigate host immune adaptations to gastrointestinal inflammation that are associated with survival. We hypothesized that the ability of the host to co-exist with large numbers of bacteria in the gastrointestinal tract requires either a dampened systemic inflammatory response or the ability to tolerate inflammation. To test this, we compared the host immune responses in super-shedder and moderate-shedder mice and characterized an immune state specific to super-shedders. Broadly, we found super-shedders have an activate innate inflammatory response in both the gastrointestinal tract and the systemic organs but a dampened adaptive T cell response specific to the systemic sites. Finally, we identify an unexpected host immune mechanism mediated by neutrophils that controls T_H_1 cell expansion in the super-shedder immune state.

## Results

### Super-shedder mice have high levels of neutrophils in systemic tissues

**Figure 1:**
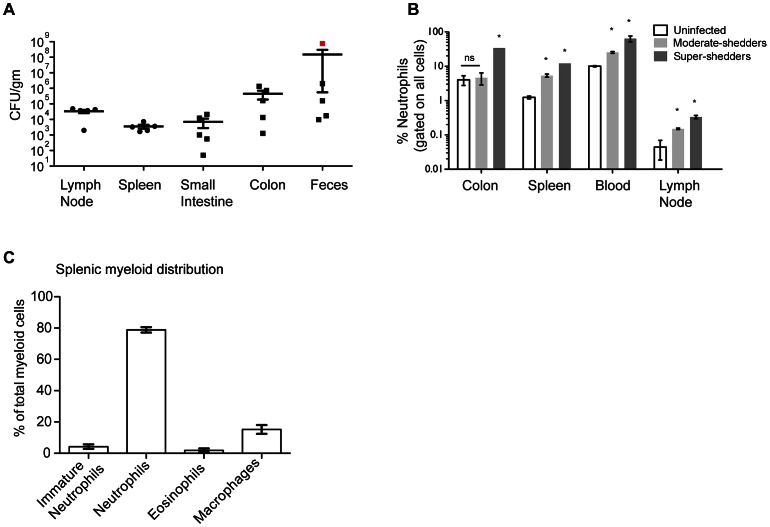
High fecal bacterial load is associated with systemic neutrophilia. All infections were conducted for 30 days unless otherwise indicated. A: Bacterial burden of indicated organs was quantified. B: Gr1^+^ cells are represented as a percentage of total cells of a single cell suspension of indicated organs. Data from at least three and a maximum of four mice per condition are shown. All differences between super-shedders, moderate-shedders and uninfected mice are significant with p<0.05 calculated using the Mann-Whitney U test, two-tailed. C: Cytospins were prepared from splenocytes of a representative super-shedder mouse infected for 30 days. A 1000 count differential was performed to identify the myeloid cell composition of the super-shedder spleen. These data are averaged across 4 mice and are representative of experiments including a total of 15 mice.

To examine the host immune response to persistent *Salmonella* infection, we first enumerated the bacterial burden in gastrointestinal and systemic tissues of mice infected with *S.* Typhimurium for 30 days. Bacterial loads in the spleen and the mesenteric lymph nodes (MLN) were tightly clustered across mice, while those in the gastrointestinal sites showed the large variation characteristic of super-shedders and moderate-shedders ([18], Figure 1A). It is known that super-shedder mice develop colitis that results in an influx of granulocytes to the colon [18]. We identified moderate and super-shedder mice as outlined in material and methods and asked if the neutrophilia extended to secondary lymphoid organs. We observed a notable increase in the frequency of Gr1^+^ (Ly6G^+^Ly6C^+^) granulocytes in the spleen, MLN, and blood (Figure 1B) of super-shedder mice compared to moderate-shedders. Super-shedder spleens were composed of 12±2.8% neutrophils; in contrast, on average, the moderate-shedder spleen contained only half that amount (5. 3±1.1% neutrophils). Histological analysis demonstrated that neutrophils constituted over 80% of splenic myeloid cells (Fig. 1C). Neutrophils were also the most numerous cell type in super-shedder blood, with 63.6±21.6% of the non-red blood cells identified as circulating neutrophils, while moderate-shedder blood contained only 25.3±3% neutrophils. When compared to uninfected mice, the levels of neutrophils in the systemic organs of moderate-shedders were significantly higher (Figure 1B). However, no significant difference in colonic neutrophil levels between uninfected and moderate-shedder mice was observed indicating that neutrophilia in the systemic sites was a stronger indicator of shedding status. Taken together, our data suggest that neutrophil levels in the systemic sites are positively correlated with gastrointestinal bacterial load.

### Splenic CD4 T cells from super-shedders display blunted T_H_1 responses

Having shown that splenic neutrophilia varied with gastrointestinal and not splenic bacterial burden, we examined whether there were associated variations in adaptive immune responses. To exploit the variation in fecal shedding, we asked what aspects of the host immune response varied with fecal bacterial load.

**Figure 2:**
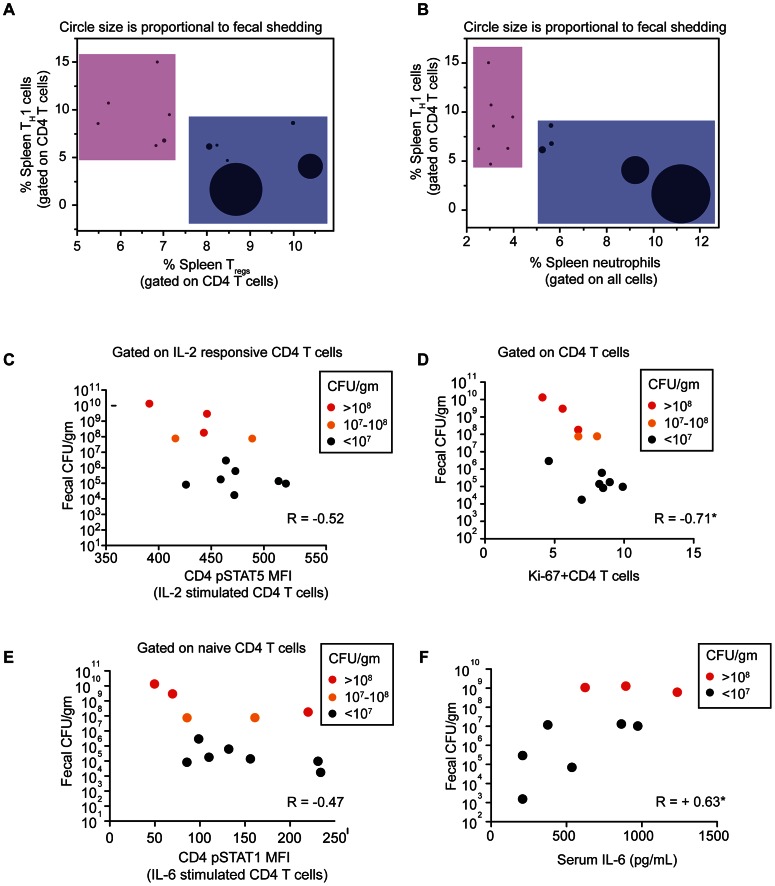
High fecal bacterial load is associated with dampened splenic T cell response. A–E: Twelve mice are represented and data is representative of 3 independent experiments with a total of 30 mice. Each circle represents an individual mouse. Asterisks indicate significant R values determined using Spearman's correlation, two-tailed. C–F: Each point represents a single mouse with red and orange dots representing super-shedders as confirmed by cecal and colonic inflammation. A,B: Tbet^+^ (T_H_1) and FoxP3^+^ (T_regs_) CD4 T cells were measured as a percentage of total CD4 T cells in the spleen. Neutrophils were measured as a percentage of total splenocytes. Splenic T_H_1 and T_regs_ were significantly negatively correlated with a R value of −0.58*. Fecal bacterial load was negatively correlated with splenic T_H_1 (R = −0.49) and significantly positively correlated with splenic T_regs_ (R = +0.65*). Finally, splenic neutrophils were significantly positively correlated with fecal bacterial load (R = +0.9*) and negatively correlated with T_H_1 cells (R = −0.43). C: Splenocytes were stimulated ex vivo for 15 minutes with 40 ng/ml IL-2. IL-2 responsive pSTAT5^+^ CD4 T cells were gated and the mean fluorescence intensity (MFI) of pSTAT5 measured. D: Ki-67^+^ cells were quantified as a percentage of total CD4 T cells and plotted against fecal bacterial burden of the mice. E : Splenocytes were stimulated ex vivo for 15 minutes with 40 ng/ml IL-6. pSTAT1 MFI of naïve CD4 T cells is represented on the y axis. F: Circulating levels of IL-6 was measured in the serum of 10 mice in a different experiment and plotted against fecal bacterial burden. Fecal shedding was significantly positively correlated with an R value of +0.63*. This was repeated twice with a total of 20 mice.

Given the importance of CD4 T cells in the host defense against *Salmonella* infection, [24] we first focused on the frequencies of two CD4 T cell subsets: T_H_1 and T_regs_. The host immune response against *Salmonella* infection requires the induction of a CD4 type 1 Helper T cell or T_H_1 immune response, involving CD4 T cells expressing the transcription factor T-bet. T_H_1 activity can in turn be controlled by regulatory T cells (T_regs_) expressing the transcription factor FoxP3. In one representative experiment, we measured these subsets in the spleens and colons of 12 mice of which five were super-shedders. This was confirmed by the levels of colonic inflammation though 2 of the 5 mice were shedding between 10^7^ and 10^8^ cfu/gm. The infected mice clustered into two distinct groups with 5 of the 7 moderate-shedders clustered together with fewer T_regs_ and more T_H_1 cells while 4 out of the 5 super-shedders were in a cluster that contained fewer T_H_1 and more T_regs_ (pink box vs. blue box, Figure 2A). The percentage of T-bet^+^ CD4 T cells (T_H_1) cells in the spleen significantly negatively correlated with fecal bacterial burden (Spearman's correlation  = −0.58) but was not significantly correlated to the splenic bacterial burden (data not shown). Additionally, this dichotomy between active and suppressive T cells was not observed in the colon (Figure S1A). Importantly, in uninfected mice there was a positive correlation between the frequencies of CD4 T cells expressing T-bet and those expressing FoxP3, indicating that the skewing in the populations of T_H_1 and T_reg_ cells is dependent on infection and is not a result of an underlying natural variation in the uninfected mouse population (Figure S1B). We found very few Rorγt-expressing CD4 T cells in persistently infected mice (data not shown). Infection-induced variation was further evidenced by the relationship between T_H_1 and neutrophil percentages in the spleens of infected mice. All 5 super-shedder mice clustered together (blue box) with high levels of neutrophils and correspondingly lower levels of T_H_1 cells. All moderate-shedders clustered on the opposite end with fewer than 5% splenic neutrophils but higher frequencies of T_H_1 cells (Figure 2B). Correspondingly, splenic neutrophilia was significantly positively correlated with fecal bacterial load (spearman's correlation R = 0.9). Importantly, splenic bacterial load did not correlate with fecal bacterial load (Figure S2A).

CD4 T cell exhaustion is a hallmark of persistent viral infections [12]–[14] so we sought to determine if these T_H_1 cells maintained antigen-responsiveness. Splenocytes from infected mice were incubated with *S.* Typhimurium-infected macrophages and the level of intracellular IFNγ levels was measured. Salmonella-specific IFNγ^+^ Tbet^+^ CD4 T cells were first detected at 8 days post-infection and expanded continuously through 30 days post-infection (Figure S3A). While culling of flagellin-specific CD4 T cells has been previously reported during *Salmonella* infection [16], we saw a steady expansion of total memory effector CD4 T cells over a time course of infection (Figure S3B). Antigen specific IFNγ production was observed in CD4 T cells across all mice, regardless of shedder status (data not shown). These data show that while super-shedder spleens have lower numbers of T_H_1 T cells relative to moderate shedders, these cells still make IFNγ in response to *Salmonella* antigen. Finally, we asked if the dampened T_H_1 response also resulted in reduced antibody production. To determine this, we measured anti-*Salmonella* antibodies in the serum of persistently infected mice and found no correlation with shedding status (Figure S2B) indicating that the blunted T_H_1 response in super-shedders did not affect antibody production.

### Splenic CD4 T cells from super-shedders have dampened responses to IL-2

Having determined that the T_H_1 cells were antigen-responsive, we further investigated the ability of CD4 T cells to respond to IL-2, a cytokine that induces T cell proliferation. The high affinity IL-2 receptor is expressed on T_regs_ and memory effector CD4 T cells; upon binding IL-2 one of the first steps initiated in the intracellular signaling cascade is the phosphorylation of STAT5 protein. Thus, the mean fluorescence intensity (MFI) level of phosphorylated STAT5 was measured in the subset of CD4 T cells that responded to IL-2 (gated on pSTAT5^+^CD4+ T cells). This metric represented the degree of cytokine responsiveness and correlated negatively with the gastrointestinal *Salmonella* burden (Figure 2C). pSTAT5 response to ex vivo IL-2 stimulation has been previously established as an indicator of T cell expansion in vivo [25]. To determine if this reduction in IL-2 responsiveness in super-shedders coincided with reduced T cell proliferation we measured the expression of Ki-67, a marker of actively proliferating cells. Consistent with decreased IL-2 responsiveness, super-shedder mice had significantly fewer Ki-67^+^ CD4 T cells in the spleen (Figure 2D). That STAT5 phosphorylation correlated inversely with the levels of bacterial shedding suggests that a feature of the super-shedder immune response involves blunting of IL-2 responsiveness. We therefore evaluated the extent to which other alterations in persistent immune responses were linked to fecal shedding status.

### Super-shedder mice have higher serum levels of IL-6 and dampened splenic CD4 T cell response to IL-6

Given the dampened IL-2 responsiveness of CD4 T cells, we asked if the ability to respond to other cytokines was also impaired. Previous work in a mouse model of septicemia showed that naïve splenic CD4 T cells have a dampened response to IL-6 compared to uninfected mice, indicated by a reduced ability to phosphorylate STAT1 in response to ex vivo IL-6 stimulation [26]. In the CD4 T cell compartment, the IL-6 pSTAT1 response is primarily restricted to naïve cells, as memory effector cells express very little IL-6 receptor (data not shown). Naïve CD4 T cell pSTAT1 responsiveness to IL-6 negatively correlated to fecal shedding levels (Figure 2E), reminiscent of the IL-2 response observed earlier. It is important to note that the IL-6 response is dampened in super-shedder mice only with respect to moderate or low shedders. Compared to uninfected mice, *Salmonella* infection induces increased IL-6 responsiveness in naïve CD4 T cells (Figure S4) but this responsiveness varied with the levels of fecal shedding. Furthermore, circulating IL-6 levels were significantly higher in super-shedder mice compared to moderate-shedders and uninfected mice (Figure 2F).

Taken together, these results reveal that across equivalently infected mice, those that developed as super-shedders are characterized by an activated innate inflammatory response with high levels of circulating IL-6 and neutrophils that is associated with a spleen-specific dampened CD4 T cell response. This dampened T cell response is characterized by a partial loss of cytokine responsiveness to IL-2 and IL-6 compared to moderate-shedder mice. Finally, in super-shedder spleens, the balance of CD4 T cell subsets supports a dampened CD4 T cell response, with fewer T_H_1 cells and more T_regs_ (Table 1).

**Table 1:** Distinct systemic immune profiles associated with fecal shedding states.

	Moderate -shedders	Super-shedders
Innate Immunity		
Serum IL-6	+	+++
Neutrophils	+	+++
Adaptive Immunity		
T_H_1 cells	+++	+
IL-2 response	+++	+
Proliferating T cells	+++	+
IL-6 response	+++	+
T_regs_	−−−	−

Summary of the results in Figures 1,2 presenting a super-shedder immune state that is distinct from the moderate-shedder immune state in the parameters mentioned. It should be noted that the plus signs indicate greater frequency of the cell type or better cytokine responsiveness with respect to uninfected mice. Thus while moderate-shedders have high levels of splenic neutrophils (+) compared to uninfected mice, they have fewer neutrophils compared to super-shedder mice (+++). Similarly, the frequency of T_regs_ decreases during infection compared to uninfected. Thus, moderate shedders have fewer T_regs_ (−−−) compared to super-shedders (−).

### Streptomycin treatment of moderate-shedders induces specific aspects of the super-shedder immune state

**Figure 3:**
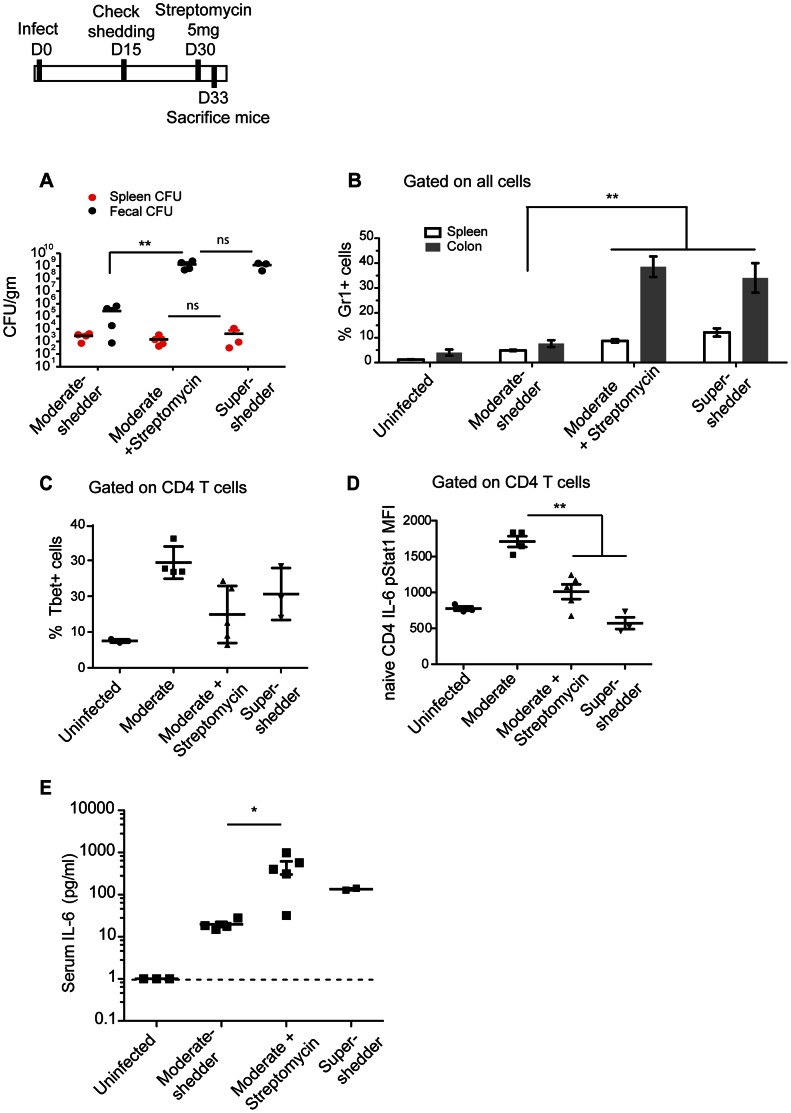
Streptomycin treatment induces the super-shedder phenotype in the gastrointestinal tract and the spleen. Mice were identified as moderate (<10^6^ cfu/gm) or super-shedder (>10^8^ cfu/gm) between day 15 and day 30 post infection. At 30 days post infection, they were treated with a single dose of 5 mg streptomycin via oral gavage and sacrificed three days after treatment. Data shown is representative of 2 independent experiments with a total of 8–10 mice in each condition. Significance was determined using a two-tailed Mann-Whitney U test with one asterisk representing p<0.05 and two representing a p<0.01. A: Bacterial burden was quantified in the spleen (red) and feces (black). B: Gr1^+^ cells are presented as the percentage of total cells in the spleen and the colon. C. Tbet^+^ T_H_1 cells were quantified as percentage of total CD4^+^T cells. D. Splenocytes were stimulated with IL-6 for 15 minutes ex vivo, fixed and permeabilized and phosphoSTAT1 expression of total CD4 T cells quantified. E. Serum IL-6 levels measured by ELISA.

Does the gastrointestinal *Salmonella* burden dictate the systemic immune profile, or does the immune response control the bacterial load? Since the numbers of bacteria in the gastrointestinal tract are correlated with specific changes in the neutrophil and CD4 T cell immune response in the spleen, we investigated whether altering the levels of *S.* Typhimurium in the gut was causal to changes in the splenic immune response. Previously it was shown that alterations of the microbiota in moderate-shedders via a single dose of streptomycin resulted in super-shedder levels of *Salmonella* in the gastrointestinal tract [18], [27]. Therefore, moderate-shedder mice were treated with an oral dose of streptomycin and their fecal shedding and splenic immune state were monitored (the *Salmonella* strain used - SL1344 - is resistant to streptomycin). Three days after streptomycin treatment, moderate-shedders shed >10^8^ *Salmonella* per gram of feces, i.e. super-shedder levels. Their splenic bacterial burden remained unchanged, as compared to untreated moderate-shedder mice (Figure 3A). Moreover, within 3 days streptomycin-treated moderate-shedders developed increased levels of neutrophils in the colon and spleen, comparable to those seen in super-shedders (Figure 3B). This was accompanied by a decrease in splenic T_H_1 cells in 3 out of the 5 streptomycin-treated mice (Figure 3C), and by one week post-treatment, all of the streptomycin-treated moderate-shedders had fewer splenic T_H_1 cells (data not shown).

Notably, streptomycin-treated moderate-shedders had increased levels of circulating IL-6 and a concomitant decrease in the ability of CD4 T cells to respond to IL-6 as measured by pSTAT1 levels (Figure 3D, Figure 3E). However, the percentage of splenic T_regs_ did not change (Figure S7A). Therefore, many aspects of the splenic super-shedder immune phenotype are induced by raising gastro-intestinal levels of *Salmonella*, although the frequency of regulatory T cells in the spleen is independently regulated.

### Neutrophil-dependent control of IL-2 responsive CD4 T cells and T_H_1 T cells

We next sought to identify which components of the host immune response control the dampened T_H_1 response observed in the spleens of super-shedder mice as compared to moderate shedders. Increased neutrophil numbers were seen in the spleen as early as four days post-infection, a time point at which *Salmonella* was undetectable outside the gastrointestinal tract (Figure S5). Since increased levels of neutrophils in the colons and spleens of super-shedder mice correlated with the dampened adaptive T_H_1 immune response, we proposed that neutrophils play a role either directly or indirectly in mediating the immune blunting.

**Figure 4:**
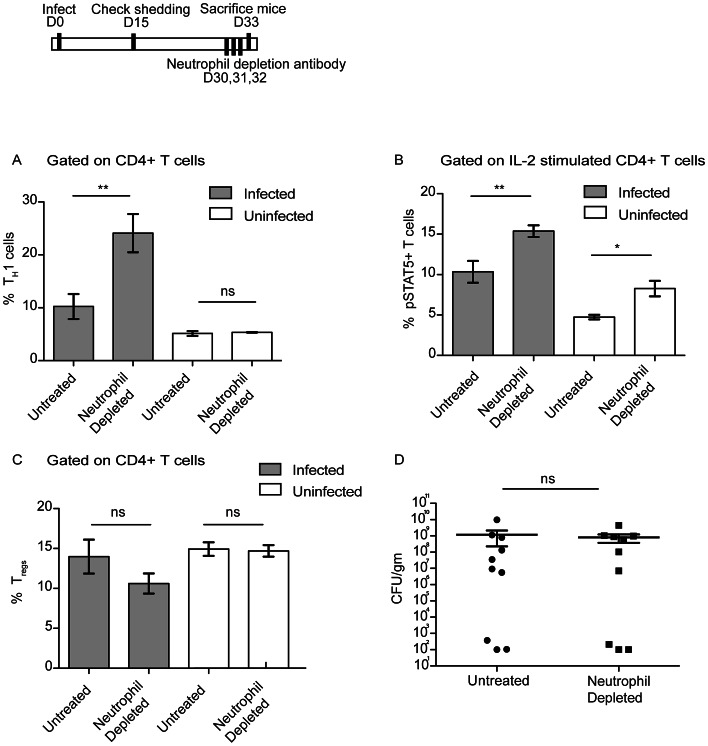
Neutrophil depletion results in increased CD4 pSTAT5 and Tbet expression. After 30 days of infection, mice were injected with 1 µg RB6 depletion antibody every day for 3 days and sacrificed on the 4^th^ day. Data shown is representative of 2 independent experiments with a total of 8–10 mice in each condition. Significance was determined using a Two-tailed Mann-Whitney U test with one asterisk representing p<0.05 and two representing a p<0.01. A: Tbet^+^ CD4 T (T_H_1) cells from splenocytes were quantified as a percentage of CD4 T cells. B: Splenocytes were stimulated ex vivo with 40 ng/ml IL-2 and fixed and permeabilized and total CD4 T cell pSTAT5 MFI measured. C: FoxP3^+^ CD4 T cells (T_regs_) were quantified as a percentage of CD4 T cells. D: Fecal pellets were collected and bacterial burden quantified.

We depleted neutrophils using the monoclonal antibody RB6, which targets cells expressing both Ly6C and Ly6G. Neutrophil depletion increased the levels of splenic T_H_1 cells from 10.2±5.8% in control mice to 24.1±8.8% in RB6-treated mice (Figure 4A). Similar results were obtained with a Ly6G-specific depletion antibody, IA8 (Figure S6A,B). An increase was observed in the frequency of pSTAT5^+^ CD4 T cells that responded to ex vivo IL-2 stimulation regardless of infection, indicating that neutrophils suppress CD4 T cell responsiveness to IL-2 (Figure 4B). Intriguingly, uninfected mice depleted of neutrophils also showed a similar increase in pSTAT5^+^ CD4 T cells, but without T_H_1 expansion. This indicates that the IL-2/pSTAT5 response may be an intermediate step to T_H_1 expansion and that T_H_1 biasing occurs only in the context of infection. The percentage of splenic T_regs_ in infected and uninfected mice did not statistically change upon neutrophil depletion, implying that the increase in IL-2 responsive CD4 cells was not due to T_reg_ reduction (Figure 4C). In addition, RB6-treated mice had significantly higher bacterial burdens in the spleen compared to control mice (Figure S6C). This demonstrates that neutrophils are necessary to control splenic infection, and that the increased T_H_1 response is unable to compensate for neutrophil depletion. Surprisingly, there was no difference observed in fecal bacterial burden (Figure 4D) suggesting that there might be an organ specific function for neutrophils in persistent *Salmonella* infection. Taken together, the results indicate that high levels of neutrophils in the spleen are necessary for both dampened IL-2 responsiveness and a reduction in the levels of of T_H_1 cells.

**Figure 5:**
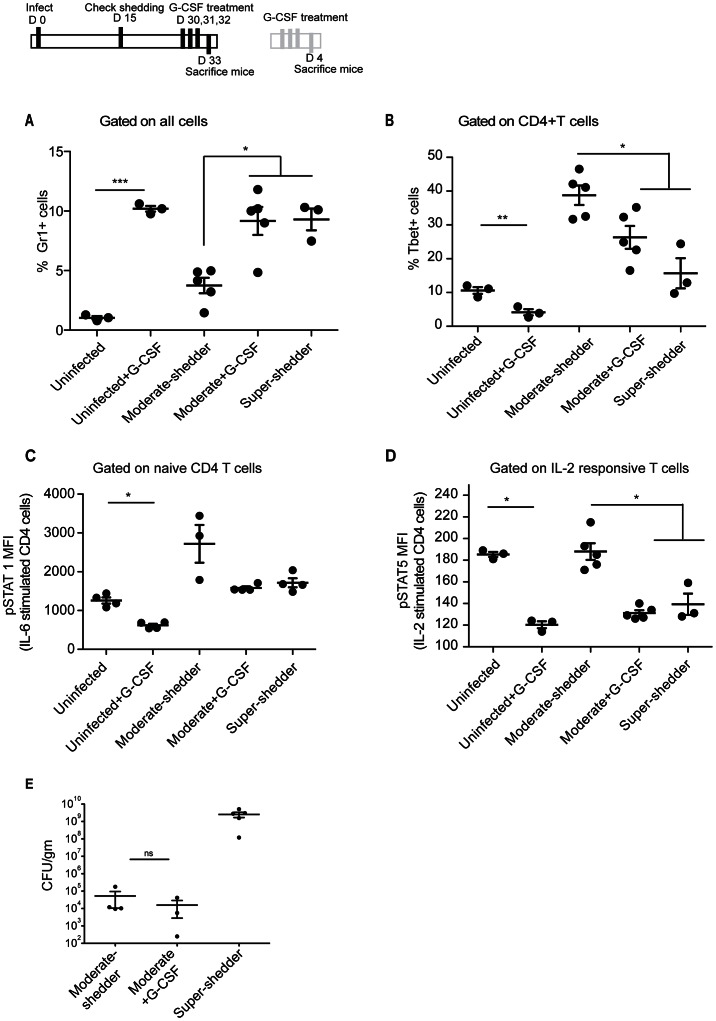
G-CSF treatment mimics the splenic super-shedder immune phenotype. Mice were identified as moderate (<10^6^ cfu/gm) or super-shedder (>10^8^ cfu/gm) between day 15 and day 30 post infection. Moderate shedders were injected with 1 µg G-CSF per day for three days and sacrificed on the 4^th^ day. Data shown is representative of 2 independent experiments with a total of 8–10 mice in each condition. Significance was determined using a two-tailed Mann-Whitney U test with one asterisk representing p<0.05 and two representing a p<0.01. A: Splenic Gr1^+^ cells are represented as a percentage of total cells in G-CSF- and PBS-treated control mice. B: Tbet^+^ T_H_1 cells from splenocytes were quantified as a percentage of CD4 T cells. C, D: Splenocytes were stimulated ex vivo for 15 minutes with 40 ng/ml of IL-6 and IL-2 and total CD4 T cell pSTAT1 and pSTAT5 MFI measured. E: Fecal bacterial burden was quantified.

Based on the finding that neutrophil depletion induced T_H_1 expansion, we investigated whether the strong systemic neutrophil induction seen in super-shedder mice was sufficient for limiting the T_H_1 response. Moderate-shedder and uninfected mice were injected with G-CSF for 3 days to induce granulopoiesis. G-CSF treatment increased splenic neutrophil levels to those observed in the spleen of super-shedder mice (Figure 5A). Importantly, G-CSF treatment of moderate-shedder mice led to a concomitant decrease in the frequency of splenic T_H_1 cells (Figure 5B). However, G-CSF treatment did not influence the frequency of T_regs_ in the spleens of moderate-shedder or uninfected mice (Figure S7B). Histological analysis of the spleen and bone marrow of G-CSF-injected mice revealed that the granulocytes induced were primarily neutrophils. Additionally, there was a significant increase in the levels of immature neutrophils in the bone marrow of G-CSF-treated moderate-shedders compared to untreated mice (Figure S8A–C).

Since G-CSF treatment caused a reduction in splenic T_H_1 cell frequencies, we assayed whether cytokine responsiveness of CD4 T cells was also blunted. Compared to untreated moderate-shedders, naïve CD4 T cells from G-CSF-treated moderate-shedders displayed a dampened pSTAT1 response to IL-6 stimulation similar to that observed in super-shedders (Figure 5C). This was finding was in alignment with the trend towards increased levels of serum IL-6 in G-CSF-treated moderate-shedders (Figure S9). G-CSF treatment also significantly dampened IL-2-mediated induction of pSTAT5 in CD4 T cells that responded to IL-2 (Figure 5D). Furthermore, uninfected mice treated with G-CSF demonstrated dampened responses to IL-2 and IL-6, indicating that neutrophil-mediated control of IL-2 induced pSTAT5 and IL-6 induced pSTAT1 responses are independent of infection (Figure 5C, 5D). These data suggested that neutrophils might suppress T_H_1 expansion via the IL-2/pSTAT5 pathway. Treatment with G-CSF did not increase fecal bacterial load (Figure 5E). Importantly, in *ex vivo* experiments, G-CSF induced STAT5 activation in granulocytes but not CD4 T cells (data not shown), indicating that G-CSF does not act directly on T cells.

### G-CSF administration suppresses IL-2 mediated T_H_1, but not T_reg_, expansion

**Figure 6:**
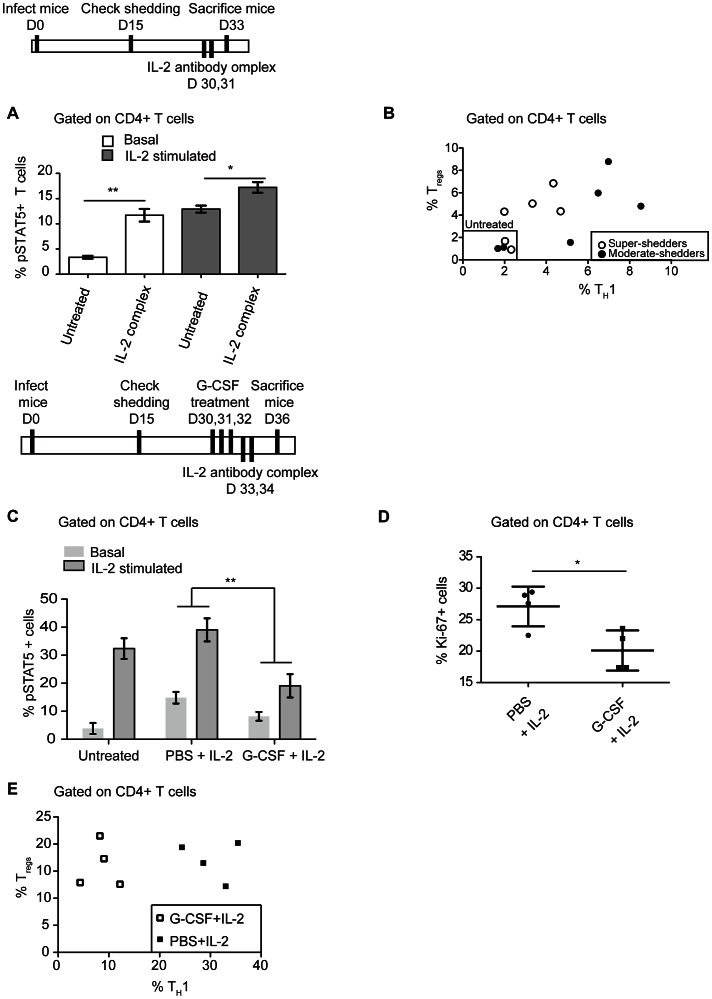
G-CSF treatment mimics the super-shedder phenotype by inhibiting IL-2 mediated T_H_1 expansion. A,B: Mice were identified as moderate (<10^6^ cfu/gm) or super-shedder (>10^8^ cfu/gm) between day 15 and day 30 post infection. After 30 days of infection, mice were injected with 15 µg IL-2 antibody complexed with 4.5 µg IL-2 cytokine in a 15 minute incubation period at room temperature. Data shown is from a single experiment with a total of 12 mice, 4 untreated and 8 treated. A: Splenocytes were fixed and permeabilized and pSTAT5 MFI quantified in all CD4 T cells. B: T_H_1 and T_reg_ levels were expressed as a percentage of total splenic CD4 T cells. Untreated mice (in square) showed no expansion and IL-2 antibody complex- treated mice included both super-shedders (open circles) and moderate-shedders (filled circles). IL-2 mediated T_H_1 expansion was significantly lower in super-shedder mice (p = 0.02), calculated using a one-tailed Mann Whitney U test. C, D, E: Moderate shedders were treated with either G-CSF or PBS for 3 days and then injected with IL-2 antibody complex on the 4^th^ day. Mice were sacrificed on the 6^th^ day, 36 days post-infection. Data shown is representative of 2 independent experiments with a total of 8 mice in each condition. Significance was determined using a two-tailed Mann-Whitney U test with one asterisk representing p<0.05 and two representing p<0.01. C: Splenocytes were stimulated ex vivo with 40 ng/ml IL-2 for 15 minutes and fixed and permeabilized and the frequency of pSTAT5^+^ CD4 T cells was quantified. D: Splenocytes were fixed and Ki-67^+^ CD4 T cells were quantified as a percentage of total CD4 T cells. E: T_H_1 and T_reg_ levels were expressed as a percentage of total splenic CD4 T cells in G-CSF treated mice (open squares) and control mice (closed squares). T_H_1 cells were significantly lower in the G-CSF pretreated mice (p = 0.02) calculated using a two-tailed Mann Whitney U test.

Having observed that G-CSF mediated neutrophilia dampens IL-2 responsiveness across the CD4 T cell population; we next investigated whether T_H_1 and T_reg_ CD4 T cell subsets differed in their IL-2 responsiveness with functional consequences. Both T_regs_ and T_H_1 cells activated by infection induce phosphorylation of STAT5 in response to IL-2 (Figure S7C). Previous studies have shown that IL-2 antibody complexed with IL-2 cytokine (hereafter referred to as IL-2 antibody complex) can induce expansion of T_regs_ in uninfected mice [28], [29]. When S. Typhimurium-infected mice were treated with IL-2 antibody complex, we observed expansion of both T_regs_ and T_H_1 cells (Figure 6B). This was accompanied by an increase in the number of pSTAT5^+^ total CD4 T cells both before (basal) and after *ex vivo* IL-2 stimulation (Figure 6A). Furthermore, IL-2 mediated T_H_1 expansion was significantly greater in moderate-shedders than super-shedders (p<0.05). These results indicate that high levels of gastrointestinal *Salmonella* burden and neutrophilia may be associated with an impairment in the ability of splenic T_H_1 cells to undergo IL-2 mediated proliferation.

**Figure 7:**
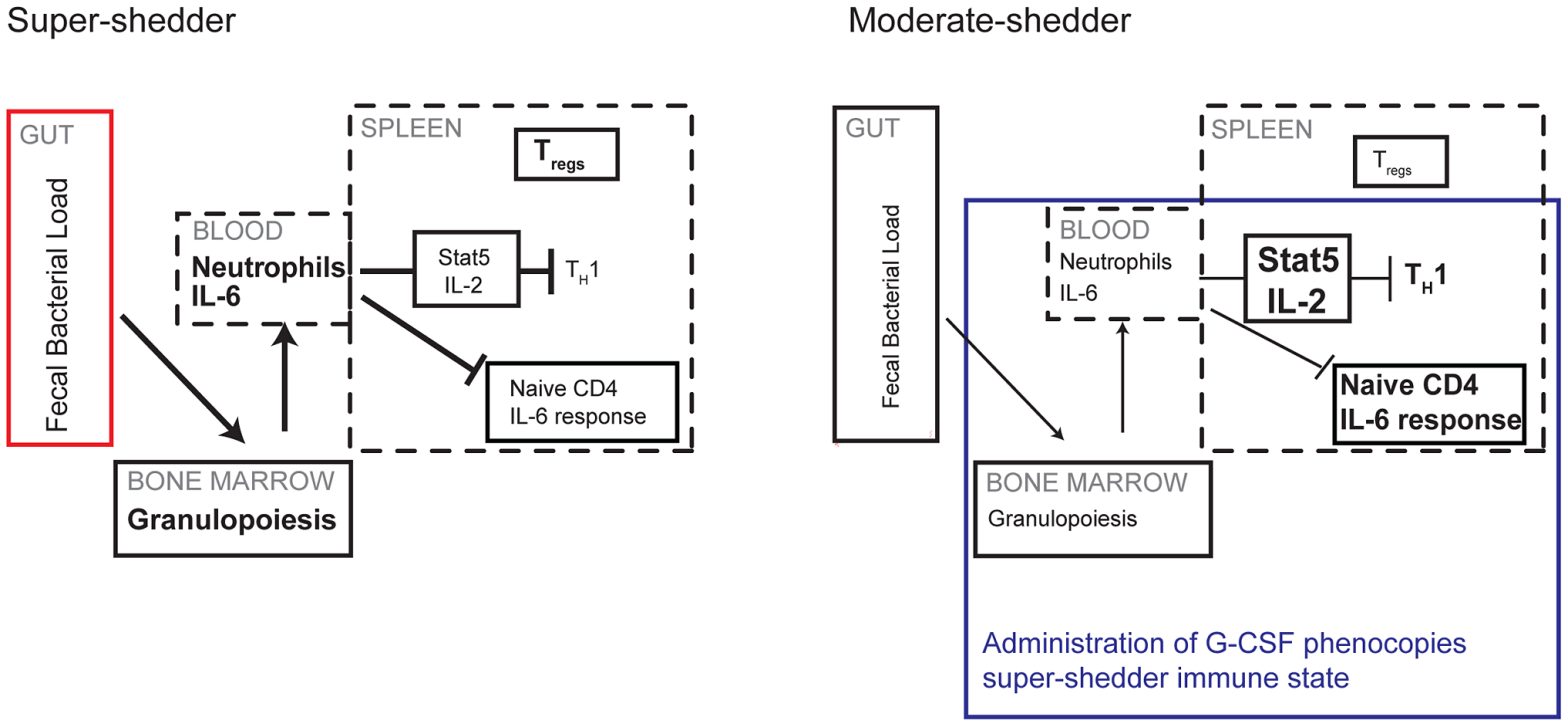
Neutrophils control the dampened systemic T_H_1 response of super-shedders. In super-shedders, high levels of *Salmonella* in the gastrointestinal tract induce systemic neutrophilia with extensive granulopoiesis occurring in the bone marrow, resulting in elevated neutrophil counts in the blood, GI tract and spleen. This is accompanied by dampened IL-2 and IL-6 cytokine responsiveness in splenic CD4 T cells, fewer T_H_1 cells and more T_regs_. In moderate shedders, low levels of gastrointestinal *Salmonella* induce very few neutrophils systemically, leading to an active IL-2 and IL-6 response with increased T_H_1 cells and fewer T_regs_. Induction of granulopoiesis in moderate-shedders results in the super-shedder immune phenotype with the exception of T_regs_, which remain unchanged.

Our findings that both gastrointestinal *Salmonella* and G-CSF-mediated neutrophilia are associated with dampened IL-2 responsiveness in T_H_1 cells, suggest that neutrophil levels influence the ability of T_H_1 cells to expand. To investigate this, moderate-shedders were treated with G-CSF for 3 days, then subsequently administered IL-2 antibody complex for another 2 days, to determine the effect of neutrophilia on T cells expansion. After IL-2 antibody complex treatment, moderate-shedders pretreated with G-SCF had lower levels of both basal and IL-2 responsive pSTAT5^+^ CD4 T cells compared with mice that were not administered G-CSF (Figure 6C). This loss of IL-2 responsiveness correlated with significantly fewer Ki-67^+^ CD4 T cells (Fig. 6D), indicating that G-CSF treatment inhibited the ability of CD4 T cells to proliferate in response to IL-2. However, this inhibition was specific to T_H_1 cells, as G-CSF did not affect T_reg_ expansion in response to IL-2 antibody complex (Figure 6E). This result is consistent with our previous finding that only T_H_1 cells and not T_regs_ expanded upon neutrophil depletion (Figure 4A, 4C). Thus, CD4 T cells in G-CSF treated moderate-shedders recapitulate the super-shedder phenotype. Taken together, we show that treatment of moderate-shedders with G-CSF is sufficient to recapitulate specific aspects of the super-shedder immune response (Figure 7).

## Discussion

We have described here a unique immune phenotype in the spleen that is linked to the ability of an enteric bacterial pathogen to replicate to high numbers in the gastrointestinal tract and thus transmit to a new host. One interesting aspect of this phenotype is that the immune state in the spleen is associated with the levels of bacteria in a distal site (the gastrointestinal tract) rather than local bacterial burden. Previous work has shown that the gastrointestinal commensal microbiota can activate antigen-presenting cells, which go on to drive adaptive immunity in distal sites such as the lung [30]. However, to the best of our knowledge, this is the first report of a splenic immune phenotype specifically associated with gastrointestinal pathogen load and inflammation.

The super-shedder immune phenotype is composed of a highly inflammatory response in both gastroinestinal and systemic sites (evidenced by neutrophilia and serum IL-6) but a dampened adaptive T cell immune response specific to the systemic sites. The high levels of circulating IL-6 and moderate to severe colonic inflammation seem at odds with the absence of weight loss or malaise observed in these mice. The blunted splenic CD4 T cell response, dampened cytokine responsiveness and increased levels of regulatory T cells might be instrumental in the tolerance of the inflammatory environment that is the super-shedder gut. In persistent bacterial infections, this might provide an opportunity for the host to suppress the long-term inflammatory effects of the adaptive T cell response while still controlling pathogen load via neutrophil recruitment.

The molecular mechanism behind neutrophil recruitment in persistent *Salmonella* infection is yet to be identified. Gastrointestinal *Salmonella* is sufficient to induce granulopoiesis and systemic neutrophilia. Neutrophils were observed in the spleen at four days post infection, a time point when Salmonella was not detected in systemic sites but was present in the gut. Previous studies have demonstrated a role for IL-17 mediated neutrophil induction [31]. IL-17 is secreted by multiple cell types including Rorγt expressing CD4 T cells or T_H_17 cells which have also been shown to play a role in acute *Salmonella* infection [32], [33]. However, we found very few Rorγt+ CD4 T cells in the spleen of persistently infected mice (data not shown). Additionally we did not detect IL-17 in serum or spleen supernatant of these mice indicating that the T_H_17 or IL-17 response does not play a role in persistent *Salmonella* infection (data not shown).

Two interventions shifted moderate shedders towards the super-shedder immune phenotype – streptomycin induction of gastrointestinal bacterial expansion and G-CSF mediated neutrophilia. In both instances, the cytokine signaling profiles and T_H_1 levels recapitulated those of natural super-shedders. However, a concordant increase in T_regs_ was not observed. This suggests that the increased T_reg_ levels observed in super-shedders are regulated by a mechanism independent of bacterial load or neutrophil levels. Indeed, the co-expansion of T_regs_ and T_H_1 cells is decoupled in super-shedder mice where IL-2 mediated T_H_1 but not T_reg_ expansion is dampened. It was surprising to find that short-term treatment with G-CSF was sufficient to recapitulate this phenotype. The mechanism behind this and other aspects of the super-shedder immune state remain unknown, as do the bacterial and host effectors required to establish this immune phenotype.

The mechanism by which induction of granulopoiesis inhibits the T_H_1 response, both in T-bet expression and IL-2 mediated expansion is unclear. It will be important to determine whether this mechanism is mediated by cell-cell contact or through cytokine secretion. Anti-inflammatory neutrophil populations which secrete IL-10 in response to Gram-negative bacteria have been previously described [34]. However, we were unable to detect IL-10 in supernatants of cultured neutrophils isolated from moderate and super-shedder mice. Additionally, co-administration of IL-10 receptor-blocking antibody along with G-CSF did not prevent the dampening of the T_H_1 response of infected mice treated with G-CSF (data not shown). One of the notable findings of this study was that T_regs_ are not suppressed in a similar manner to T_H_1 cells. Previous work has shown that T_regs_ lose potency in the later stages of persistent *Salmonella* infection [17]. Our data indicated that T_regs_ from mice infected for 30 days were capable of IL-2-mediated expansion. However, the suppressive potency of these cells was not analyzed and it is possible that the expanded population is not capable of suppressing T cells.

While induction of granulopoiesis was sufficient to induce aspects of the super-shedder systemic immune network in moderate-shedder mice, no differences were seen in fecal shedding. We suspect that this was because the persistent *Salmonella* infection had already been established. The only factors shown to induce super-shedder status so far have been antibiotic-mediated ablation of the gut microbiota or neutralization of IFNγ cytokine [18], [23], [27].The immune processes pivotal in the establishment of the super-shedder state may take place at the onset of infection. It should also be noted that G-CSF induction only mimicked the super-shedder phenotype in the spleen. The colonic and cecal inflammation typically seen in super-shedders did not develop in G-CSF-treated moderate-shedders (data not shown). It is possible that either a longer G-CSF treatment or *Salmonella*-specific induction of host chemokine responses are required for sustained gastrointestinal neutrophilia associated with gastrointestinal pathology.

As noted, the systemic super-shedder immune phenotype is primarily one of an active inflammatory response and a dampened T_H_1 response. However, these T_H_1 cells are not anergic, as they retain the ability to recognize *Salmonella* antigen and secrete IFNγ in response. The serum levels of IFNγ were elevated by four days post-infection and remained consistently high during the first month of infection (Figure S3A). Since the cytokine levels were elevated before the T cell response was initiated, these data suggest that antigen-specific CD4 T cells may not be the primary source of serum IFNγ during infection. It is likely that the source of the early IFNγ is activated macrophages or monocytes. This is further supported by our observation that serum IFNγ did not correlate with fecal shedding and was not a part of the super-shedder immune phenotype. *Salmonella*-induced blunting of flagellin-specific T cells has been previously reported [16], however, while we did not investigate the clonality of the CD4 T cell population, we saw a steady expansion of antigen-specific, IFNγ producing, memory effector CD4 T cells (Figure S3).

This study describes a distinct splenic immune signature in the *Salmonella* carrier state responsible for transmission. These results might have important implications for identification of *S.* Typhi carriers. For instance, current serological methods do not distinguish between carriers and people who have cleared the infection [35], [36]. While Typhi infection induces a neutrophillic inflammatory response, clinical reports on typhoid patients have only described a mild transient granulocytosis [37]–[39] and neutrophils are typically not found in stool samples of *S.* Typhi patients [40]. It remains unknown if any variation in neutrophil frequency was observed in typhoid patients and if this correlates with carrier status. For example, serum IL-6 levels are elevated in patients with typhoid and have been correlated with prolonged fever [41]. However, in these studies and others, immune correlates with fecal shedding are difficult to obtain. A study on 13 long-term typhoid carriers actively shedding Typhi demonstrated a wide variance in antibody titers in accordance with our data in mice infected with *S*. Typhimurium ([9], Figure S2B). In other animal models, studies in *S*. Typhimurium-infected swine uncovered positive correlations between early fecal shedding of salmonella (within the first week of infection) with increased neutrophil numbers and high serum IFNγ [42]. Identifying immune correlates that are associated with active shedding of *Salmonella* by the host would help determine biomarkers for screening of Typhoid carriers.

Interestingly, there is evidence for an immune phenotype associated with human *S.* Typhi carriers. Transcriptional profiling of a cohort of acute, convalescing and recovered typhoid patients uncovered specific neutrophil and lymphocyte gene expression sets associated with each of those stages. Specifically, while 50% of the convalescing patients had a gene expression signature indistinguishable from healthy controls, 25% of the patients showed a distinct gene expression pattern in multiple cell types that were more similar to those of newly admitted patients despite being collected 9 months after treatment. This dataset supports the possibility of a long-term alteration in immune response in a subset of S. Typhi patients [43]. The results presented here are a step toward the definition and practical identification of immune states associated with high levels of *Salmonella* fecal shedding and transmission. That this is mediated by a novel neutrophil-dependent mechanism of IL-2- mediated T_H_1 blunting, suggests novel disease management approaches both for individuals and human communities where *Salmonella* is endemic.

## Materials and Methods

### Ethics statement

All animal experiments were performed in accordance with Stanford University's Institutional Animal Care and Use Committee and NIH Guidelines for Euthanasia of Rodents Using Carbon Dioxide. All animal experiments were approved by Stanford University's Administrative Panel on Laboratory Animal Care (A-PLAC). Stanford University Animal Welfare Assurance Number: A3213-01. Protocol ID 12826. All animals were housed in a centralized research animal facility, fully staffed with trained personnel and accredited by the Association of Assessment and Accreditation of Laboratory Animal Care International (AAALAC). Mice were monitored daily; mice displaying signs of pain, distress (hunched posture, lethargy, ruffled fur) and weight loss were euthanized humanely.

### Bacterial strains and growth

*Salmonella enterica* serovar Typhimurium wild type strain SL1344 was used for all infections. This strain is resistant to streptomycin. The bacteria were grown, shaking, at 37°C overnight in Luria-Bertani (LB) broth containing 200 µg/mL streptomycin. Bacteria were spun down and washed with phosphate buffered Saline (PBS) before being resuspended into the desired concentration for infection. For macrophage infection, bacteria were diluted to the desired concentration and pipetted onto the cells.

### Mice

Female 129x1/SvJ mice were obtained from The Jackson Laboratory and infected when they were 7–9 weeks old. Food and bedding were changed once a week by the Stanford Animal Facility and access to food and water was unlimited. For infections, food but not water was removed 12–16 hours ahead. Mice were infected orally, drinking 10^8^ CFU in 20 µL PBS from a pipette tip. For the streptomycin treatment, the antibiotic was delivered orogastrically with 5 mg streptomycin (Sigma Aldrich, S6501) dissolved in 100 µL PBS. Where indicated, infected mice were identified as super-shedders (fecal shedding >10^8^ cfu/gm) and moderate-shedders (fecal shedding <10^6^ cfu/gm) at 15 days post infection.

### Feces collection and culture

To determine fecal bacterial load, fresh fecal pellets were collected by placing individual mice in sterile isolation chambers until 2–4 pellets were excreted. These were weighed and placed in 500 µL PBS. Pellets were resuspended via vortexing and CFUs were determined by plating log dilutions on LB agar plates containing 200 µg/mL streptomycin.

### Identifying shedding status

Fecal CFU was checked at 2–4 timepoints between 15 and 30 days post infection. Mice that consistently shed <10^6^ cfu/gm at all times were identified as moderate-shedders while mice that shed >10^8^ cfu/gm at all times were identified as super-shedders. Infected mice remained with their original cage mates even after determination of shedding status. Super-shedder status was also confirmed by verifying colonic and cecal inflammation after sacrifice of the animal.

### Organ bacterial loads

Individual organs were collected, weighed and homogenized in 1 mL of PBS and log dilutions plated onto LB agar containing 200 µg/mL streptomycin.

### Single cell suspensions

Spleens from mice were mechanically dissociated into single cell suspensions in RPMI (Gibco, 11875) media with 10% fetal bovine serum (FBS) (Gibco, 26140) using glass slides. Spleens weighing less than 0.25 gm were suspended in a total volume of 10 mLs, those between 0.25–0.4 gm in a total volume of 15 mLs while spleens weighing more than 0.4 gm were resuspended in total of 30 mLs. Mesenteric lymph nodes were dissociated into single cell suspensions using a motorized pestle into 1 mL (Kontes, K749540-0000). All single cell suspensions were then filtered through a 70 µm cell strainer (BD Falcon, 352350). Blood was collected via cardiac puncture into a syringe containing 100 µL Heparin (BD, 366480). Samples were spun down for 10 minutes at 8000 rpm and serum removed and stored at −80°C. The cell pellet was then resuspended in 1 mL PBS. The blood sample was then treated with ACK buffer (0.15 M NH_4_Cl, 10 mM KHCO_3_, 0.1 mM Na_2_-EDTA, pH 7.4) to lyse red blood cells as described in [44]. Colons were isolated, opened, cleaned and washed four times with PBS. Colonic tissue was subsequently cut into 5–10 mm pieces and added to 10 mLs of RPMI containing 10% FBS, 0.5% Hepes (Sigma, H3375), 0.1% β Mercaptoethanol (Sigma, M3148) as well as the following enzymes at 1 mg/mL: Collagenase Type 1A (Sigma C9891), DNase I (Roche. 10104159001), Trypsin Inhibitor Type 1-S (Sigma, T6522). The tissue was gently agitated every 15 minutes and incubated for an hour at 37°C. The cells were then passed through a 70 µm filter and spun down at 1500 rpm. Leukocytes were then isolated from the colonic cells using CD45+ microbeads (Miltenyi Biotec, 130-052-301) as described in the product datasheet. All cells were then fixed with 1.6% paraformaldehyde (Electron Microscopy Sciences,15710) for 10 minutes at room temperature, washed twice with FACS buffer (PBS containing 0.5% BSA and 0.02% sodium azide). Cells were then either stored in methanol or permeabilized with saponin and stained as described in the flow cytometry section.

### Stimulations of splenocytes

Cytokine stimulations were conducted as described in [26]. Briefly, single cell suspensions of splenocytes were recovered for 30 minutes at 37°C. Splenocytes were then left unstimulated or stimulated with 40 ng/mL IL-2 or IL-6 (BD Biosciences) for 15 minutes at 37°C. Splenocytes were then fixed as described previously, spun down and resuspended in cold methanol. Samples were stored at −80°C until staining for flow cytometry.

### Flow cytometry

Samples stored in methanol were washed twice with FACS buffer. Cells were stained for 30 minutes with surface marker antibody cocktail comprised of Gr1-APC-Cy7, B220-PE-Texas Red, CD4-Alexa Fluor 700, CD11b-PerCP-Cy5.5, CD44 (-V500 or in-house conjugated to Pacific Orange), CD25-PE, Ki-67-Alexa Fluor 647 and CD62L-biotin (with streptavidin-quantum dot605 secondary) and phospho-Stat antibodies pStat5-Alexa Fluor 488 or pStat1-Alexa Fluor 488. All these antibodies were purchased from BD Biosciences. For measurement of transcription factors, FoxP3-PE and T-bet-Alexa Fluor 647/eFluor 660 were used (eBioscience). After staining, the cells were washed with FACS buffer and run on an LSR II flow cytometer (Becton Dickinson). Cells were acquired with DIVA software (BD Biosciences) and analyzed using FlowJo software (Tree Star). Cells were either measured as a percentage of total intact cells (determined by forward and side scatter measurements) or as a percentage of a specific cell type (e.g. total CD4 T cells). Alternatively, when measuring phospho-protein expression, median fluorescence intensity (MFI) of the cell populations was used. Staining for transcription factors was carried out using saponin permeabilization buffer (PBS containing 0.3% saponin, 0.5% BSA and 0.02% sodium azide). Cell populations were gated as described in Figure S10.

### Neutrophil depletion

Mice were injected with 1 µg each of one of two different neutrophil depletion antibodies; anti-Ly6G (clone IA8, BioXcell, BE0075-1) and anti Gr1 (clone RB6-8C5, BioXcell, BE0075-1) or PBS controls. Antibodies or PBS were administered intraperitoneally every day for 3 days and mice sacrificed on the fourth day.

### Serum cytokine levels

Serum was collected as described above and IFNγ (eBioscience, 88-8314-22) and IL-6 (BD Bioscience, 550950) levels were measured using sandwich ELISA kits.

### Serum antibody titers

ELISA plates were coated with Salmonella lysate for 1 hour at 37°c for 1 hour, then blocked with 3% Bovine Serum Albumin in PBS for 1 hour. Serum samples were then diluted 10 fold in wash buffer for a minimum of six serial dilutions. Samples were incubated for 2 hours at 37°c, washed, and incubated with Biotin conjugated anti- mouse IgG (Abcam, ab64255) for a further 2 hours at the same temperature. Plates were washed then incubated with Streptavidin conjugated Horse Radish Peroxidase (R&D,DY998) for 30 minutes, washed and incubated with TMB substrate (BD,555214) for 10 minutes, then stopped with 2N H_2_SO_4_. Titers were estimated by determining the lowest sample dilution with an optical density reading higher than undiluted serum from uninfected mice. All washes were carried out 5 times in between all steps using wash buffer consisting of 0.5% Tween in PBS.

### Cytologic evaluation and differential counting

Spleens were processed as described above and bone marrow was harvested by flushing a single tibia with 1 mL of RPMI containing 10% FBS. Cytologic specimens were prepared from single-cell suspensions of harvested bone marrow and spleen via concentration of the suspensions using a cytocentrifuge (Shandon Cytospin 4, Thermo Fisher Scientific, Waltham, MA). Slides were fixed and stained with modified Wright-Giemsa (Accustain, Sigma-Aldrich, St. Louis, MO). All slides were reviewed in a blinded fashion by a board-certified veterinary clinical pathologist. 1000-cell differential counts were performed with all myeloid and erythroid cells categorized as either proliferative or maturing (post-mitotic). For example, proliferative neutrophilic cells include myeloblasts, promyelocytes and myelocytes; maturing neutrophilic cells include metamyelocytes, band and segmented neutrophils. Neutrophils, eosinophils, macrophages, lymphocytes and plasma cells were further separated into individual categories.

### G-CSF treatment

Pegylated G-CSF (GenScript, Z00393-50) was resuspended in PBS at a final concentration of 1 mg/mL and injected intraperitoneally at a dosage of 1 ug/mouse. Control mice were injected with 100 ul of PBS intraperitoneally. Mice were injected for three days consecutively and sacrificed on the fourth.

### Antigen-specific IFNγ secretion

Bone marrow-derived macrophages were prepared as previously described [45]. Five days after thawing, macrophages were plated at 2.5×10^5^ cells per well in a 24 well dish (Corning). The cells were then infected at a multiplicity of infection of 5. Three hours after infection, supernatant was removed and 500 µL of the splenocyte single cell suspension added. After three hours of incubation, the cell suspension was collected. Cells were fixed, permeabilized with saponin and stained for intracellular IFNγ and transcription factors.

### IL-2 antibody complex treatment

IL-2 antibody complex was prepared as described previously [29]. IL-2 mouse antibody (clone JES6-1, eBiosciences, 16-7022-81) was incubated with recombinant mouse IL-2 (eBiosciences, 14-8021-64) for 15 minutes before intraperitoneal injection into mice. Mice were injected for 2 days consecutively and sacrificed on the third. Control mice were injected with an equivalent volume of PBS.

### Visualizations

Bubble plots in Figures 2 A,B and Supplementary Figure 1A were constructed using JMP software (SAS software, Cary, NC). Supplementary Figure 4C was visualized using MATLAB (MathWorks, Natick, MA). All other Figures were made using Prism (GraphPad, La Jolla, CA). All statistics were calculated using Prism and a two-tailed Mann-Whitney non-parametric test of significance was used unless otherwise mentioned. Spearman's correlations values were deemed significant using a two-tailed calculation based on the number of samples.

## Supporting Information

Figure S1 **The negative correlation between T_H_1 and T_regs_ is not present in the colons of infected mice or in the spleens of uninfected mice.** Data shown is representative of two independent experiments with a total of 8–10 mice in each condition. Asterisks indicate significant R values determined using Spearman's correlation, two-tailed. A: Foxp3^+^ T_regs_ and Tbet^+^ T_H_1 cells were quantified as a percentage of total CD4 T cells in the colons of 8 infected mice. Each point represents a mouse and the size of the circle is indicative of fecal bacterial load. Colonic T_regs_ and T_H_1 were not significantly positively correlated with Spearman's R value = +0.62. B Foxp3^+^ T_regs_ and Tbet^+^ T_H_1 cells were quantified as a percentage of total CD4 T cells in the spleens of 10 uninfected mice. Spearman's R value = +0.66.
(TIF)

Figure S2 **Fecal bacterial loads do not correlate with splenic bacterial loads or with total IgG antibody titers.** A,B: Data from 12 mice represented in Figures 2A–E are shown and is representative of 3 independent experiments with a total of 30 mice. As in Figure 2, each point represents a single mouse with red and orange dots representing super-shedders as confirmed by cecal and colonic inflammation. A. Splenic CFU of Salmonella is plotted against fecal CFU. B. Serum IgG antibody titers were measured by diluting serum from infected mice in 10 fold serial dilutions and determining the lowest dilution displaying an absorbance reading higher than undiluted serum from uninfected mice.
(TIF)

Figure S3 **Steady expansion of memory effector CD4 T cell response in persistent** ***Salmonella*** **infection.** Time course of infection with 5 infected mice and 2 uninfected mice were sacrificed at the time points indicated. A: Bone marrow-derived macrophages were infected with *Salmonella* for 5 hours at a multiplicity of infection of 5, as described previously {McLaughlin, 2009 #141}. Subsequently, splenocytes from mice at the indicated time points post-infection were cultured with the infected bone marrow-derived macrophages for 3 hours. Splenocytes were subsequently stained for intracellular IFNγ and the number of Tbet^+^ IFNγ^+^ cells were quantified. On the right hand y-axis, serum levels of IFNγ was measured on indicated days. B: CD4 memory effector cells (CD4^+^ CD44^hi^ CD62L^−^) and CD4 naïve cells (CD4^+^ CD44^−^ CD62L^hi^) were quantified as a percentage of total splenic CD4 T cells at the indicated time points.
(TIF)

Figure S4 **CD4 IL-6 responsiveness is increased in infected mice compared to uninfected mice but dampened in super-shedders compared to moderate-shedders.** A, B: Splenocytes from 3 uninfected and 5 30-day infected mice were recovered and stimulated with 40 ng/mL IL-6 for 15 minutes then fixed and permeabilized. Data is representative of 3 independent experiments conducted with a total of 30 mice. A: pSTAT1 MFI of stimulated and unstimulated samples is shown. The red dot indicates the single super-shedder in the group of infected mice. B: Representative FACS plots of unstimulated and IL-6 stimulated samples from uninfected, super-shedder and moderate-shedder mice are depicted. X axis depicts CD62 Ligand expression and pSTAT1 MFI is shown on the Y axis.
(TIF)

Figure S5 **Neutrophils are an early indication of infection in the spleen, before detectable bacterial loads.** A: Bacterial burden was quantified from the spleen and feces at days indicated post-infection. On the right y-axis, the number of Gr1^+^ cells (neutrophils) from the spleen were measured at the indicated time points. On the left y-axis bacterial load is represented on a log scale. Black squares represent fecal bacterial burden and yellow squares represent splenic bacterial burden at the indicated time points.
(TIF)

Figure S6 **Tbet expression and splenic Salmonella levels are increased upon neutrophil depletion.** Persistently infected mice were injected with neutrophil depletion antibodies (RB6 or IA8) or PBS controls for three days and sacrificed on the fourth. A. Representative FACs plots of splenocytes from mice treated with two different neutrophil depletion antibodies, IA8 which targets LY6G and RB6 which targets both Ly6C and Ly6G and PBS treated controls. B,C: Data is shown from 4–6 mice per condition and the experiment was repeated twice for a total of 10–12 mice per condition. Asterisks indicate p<0.05 calculated using two-tailed Mann-Whitney U test. B. T_H_1 cells are quantified as percentage of Tbet+ CD4 T cells of total CD4 T cells in the spleen. C. Splenic bacterial burden was measured in RB6 treated and PBS control mice.
(TIF)

Figure S7 **G-CSF does not impact the T_reg_ arm of the adaptive immune response.** A: Splenocytes from uninfected, moderate-shedder and streptomycin-treated moderate-shedder mice were collected as detailed in Figure 3 and T_regs_ were quantified as a percentage of total CD4 T cells. Data is shown from 3–5 mice per condition and the experiment was repeated twice for a total of 8–10 mice per condition. B. Uninfected and 30 day infected moderate-shedder mice were injected with G-CSF for 3 days and sacrificed as described in Figure 5. T_regs_ were quantified as a percentage of CD4 T cells in the spleen. Data shown is representative of 2 independent experiments with a total of 8–10 mice in each condition. C: Single cell suspensions were collected from the mesenteric lymph nodes of infected mice and stimulated ex vivo for 15 mins with 40 ng/ml IL-2, then fixed and permeabilized. Warmer colours indicate higher pSTAT5 MFI. Data shown is from a single mouse in stimulated and unstimulated conditions. It is representative of many independent experiments with a total of 30 mice.
(TIF)

Figure S8 **G-CSF treatment of moderate-shedders alters their bone-marrow and splenic cellular composition, resulting in increased neutrophils.** A,B: Cytospins were performed on spleen (A) and bone marrow (B) samples from uninfected and infected mice with G-CSF and control (PBS) injected mice and quantified using 1000 cell differentials. Images from representative samples are shown. C: Mature and immature neutrophils were quantified as a percentage of the total cells. Asterisks indicate p<0.05 calculated using two-tailed Mann-Whitney U test and comparing to moderate-shedder or uninfected samples.
(TIF)

Figure S9 **Serum IL-6 is increased in G-CSF treated mice.** Serum IL-6 was quantified in G-CSF treated moderate shedders and control (PBS) moderate shedders. There were four mice in each condition and a one-tailed Mann-Whitney U test yielded a p value of 0.1.
(TIF)

Figure S10 **Gating strategy for flow cytometry data.** Single cell suspensions were stained with the antibodies as mentioned in the materials and methods. Cells were gated on Forward and Side Scatter to exclude debris and red blood cells. After that, a hierarchical gating cluster was employed to identify Neutrophils, B cells, CD4 T cells and T_H_1 and Tregs as depicted.
(TIF)
