# Supplementary figures and images for: The Systemic Immune State of Super-shedder Mice Is Characterized by a Unique Neutrophil-dependent Blunting of TH1 Responses
Source: PLoS Pathog. 2013 Jun 6;9(6):e1003408. doi: 10.1371/journal.ppat.1003408 (PMC3675027; doi:10.1371/journal.ppat.1003408)

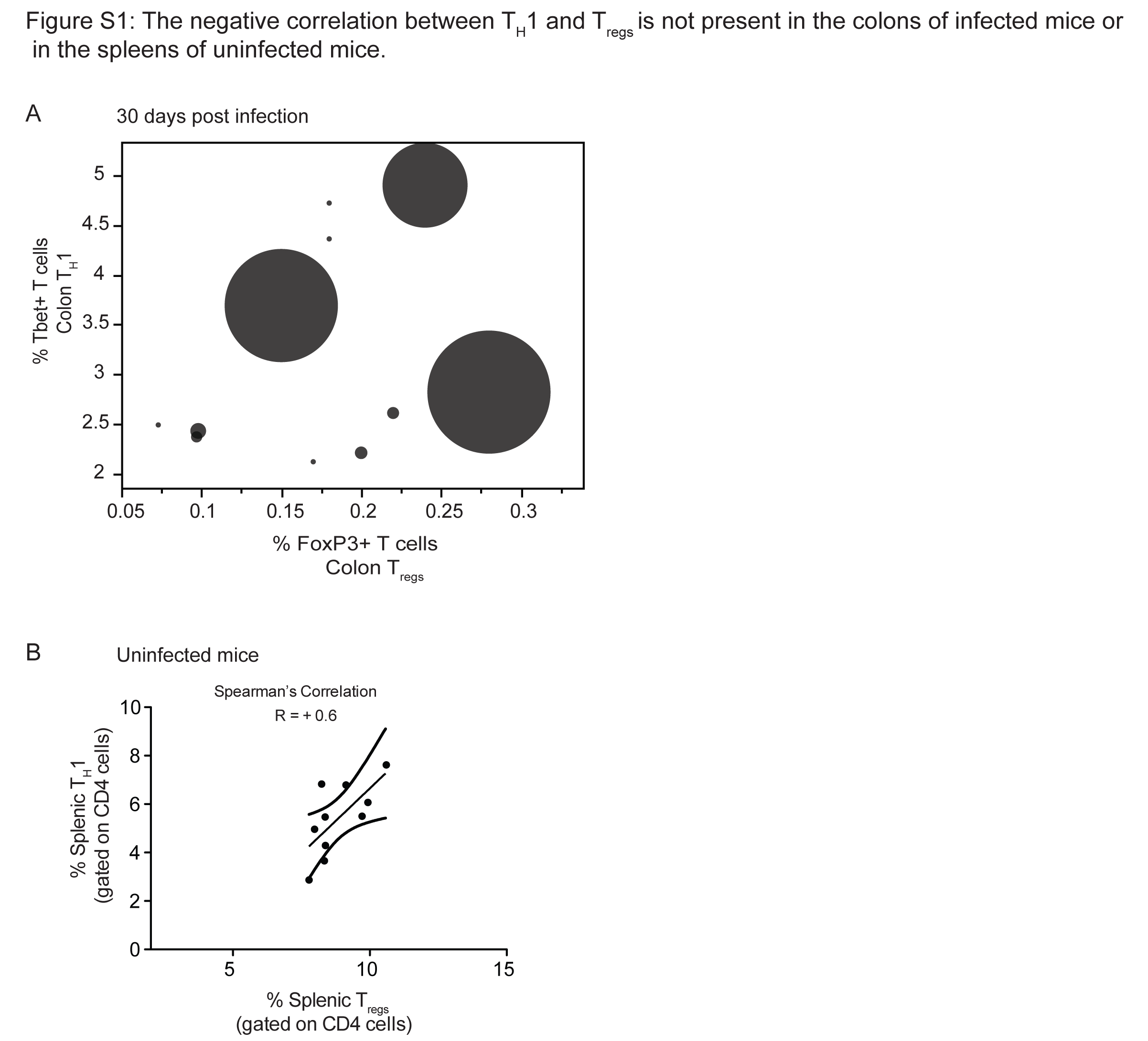

Supplement: Figure S1 — The negative correlation between TH1 and Tregs is not present in the colons of infected mice or in the spleens of uninfected mice. Data shown is representative of two independent experiments with a total of 8–10 mice in each condition. Asterisks indicate significant R values determined using Spearman's correlation, two-tailed. A: Foxp3+ Tregs and Tbet+ TH1 cells were quantified as a percentage of total CD4 T cells in the colons of 8 infected mice. Each point represents a mouse and the size of the circle is indicative of fecal bacterial load. Colonic Tregs and TH1 were not significantly positively correlated with Spearman's R value = +0.62. B Foxp3+ Tregs and Tbet+ TH1 cells were quantified as a percentage of total CD4 T cells in the spleens of 10 uninfected mice. Spearman's R value = +0.66. (TIF) [file ppat.1003408.s001.tif]

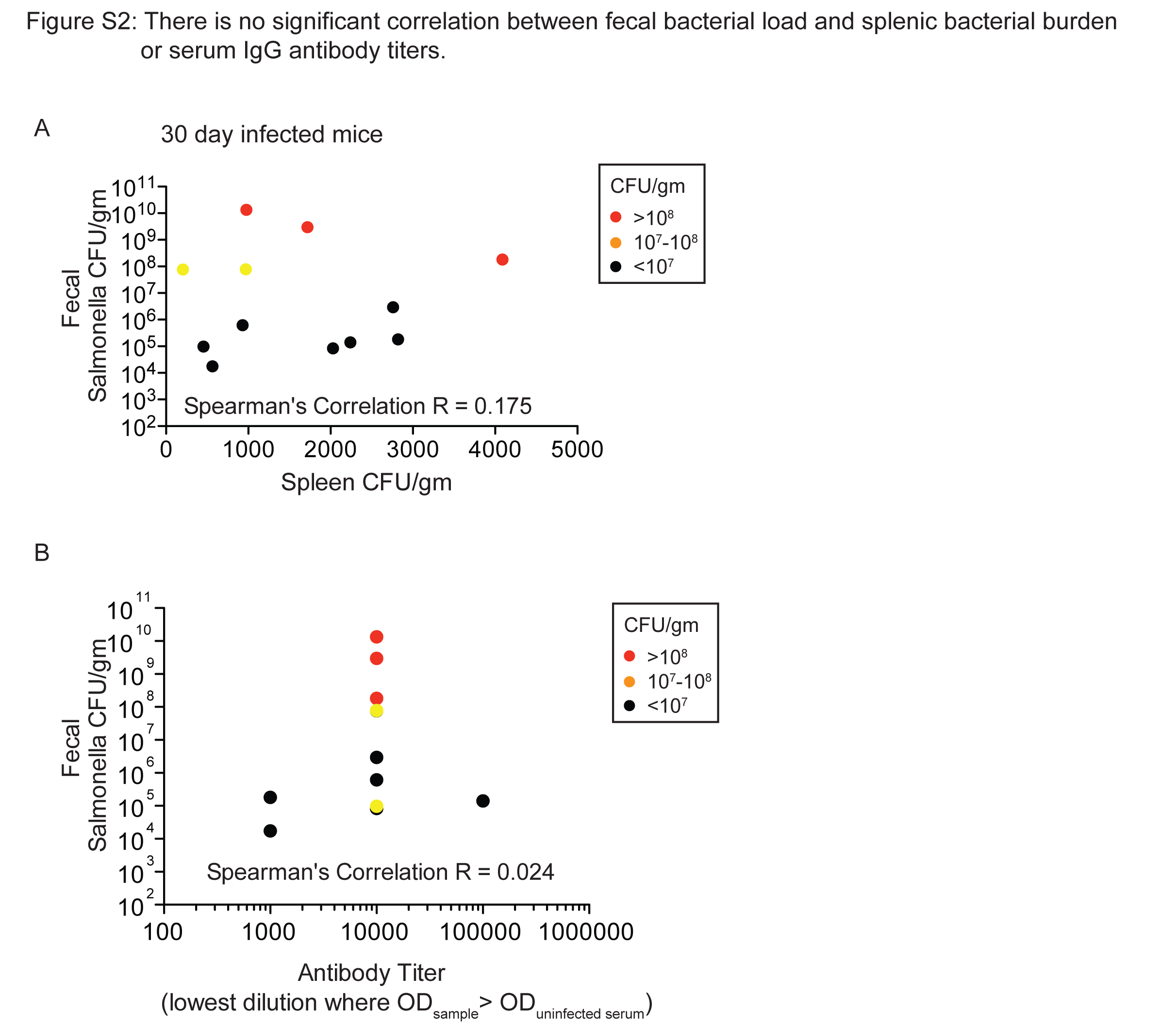

Supplement: Figure S2 — Fecal bacterial loads do not correlate with splenic bacterial loads or with total IgG antibody titers. A,B: Data from 12 mice represented in Figures 2A–E are shown and is representative of 3 independent experiments with a total of 30 mice. As in Figure 2, each point represents a single mouse with red and orange dots representing super-shedders as confirmed by cecal and colonic inflammation. A. Splenic CFU of Salmonella is plotted against fecal CFU. B. Serum IgG antibody titers were measured by diluting serum from infected mice in 10 fold serial dilutions and determining the lowest dilution displaying an absorbance reading higher than undiluted serum from uninfected mice. (TIF) [file ppat.1003408.s002.tif]

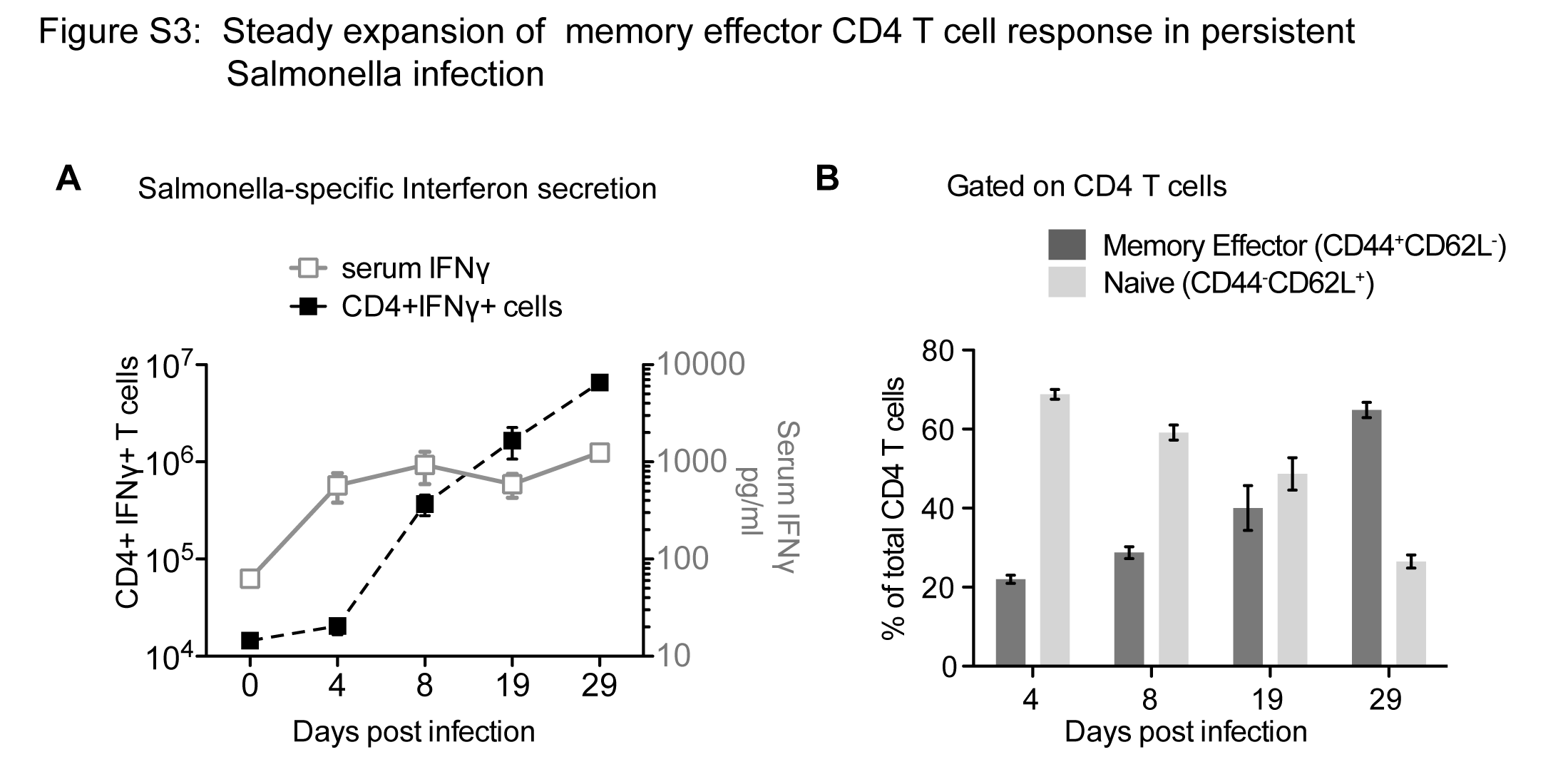

Supplement: Figure S3 — Steady expansion of memory effector CD4 T cell response in persistent Salmonella infection. Time course of infection with 5 infected mice and 2 uninfected mice were sacrificed at the time points indicated. A: Bone marrow-derived macrophages were infected with Salmonella for 5 hours at a multiplicity of infection of 5, as described previously {McLaughlin, 2009 #141}. Subsequently, splenocytes from mice at the indicated time points post-infection were cultured with the infected bone marrow-derived macrophages for 3 hours. Splenocytes were subsequently stained for intracellular IFNγ and the number of Tbet+ IFNγ+ cells were quantified. On the right hand y-axis, serum levels of IFNγ was measured on indicated days. B: CD4 memory effector cells (CD4+ CD44hi CD62L−) and CD4 naïve cells (CD4+ CD44− CD62Lhi) were quantified as a percentage of total splenic CD4 T cells at the indicated time points. (TIF) [file ppat.1003408.s003.tif]

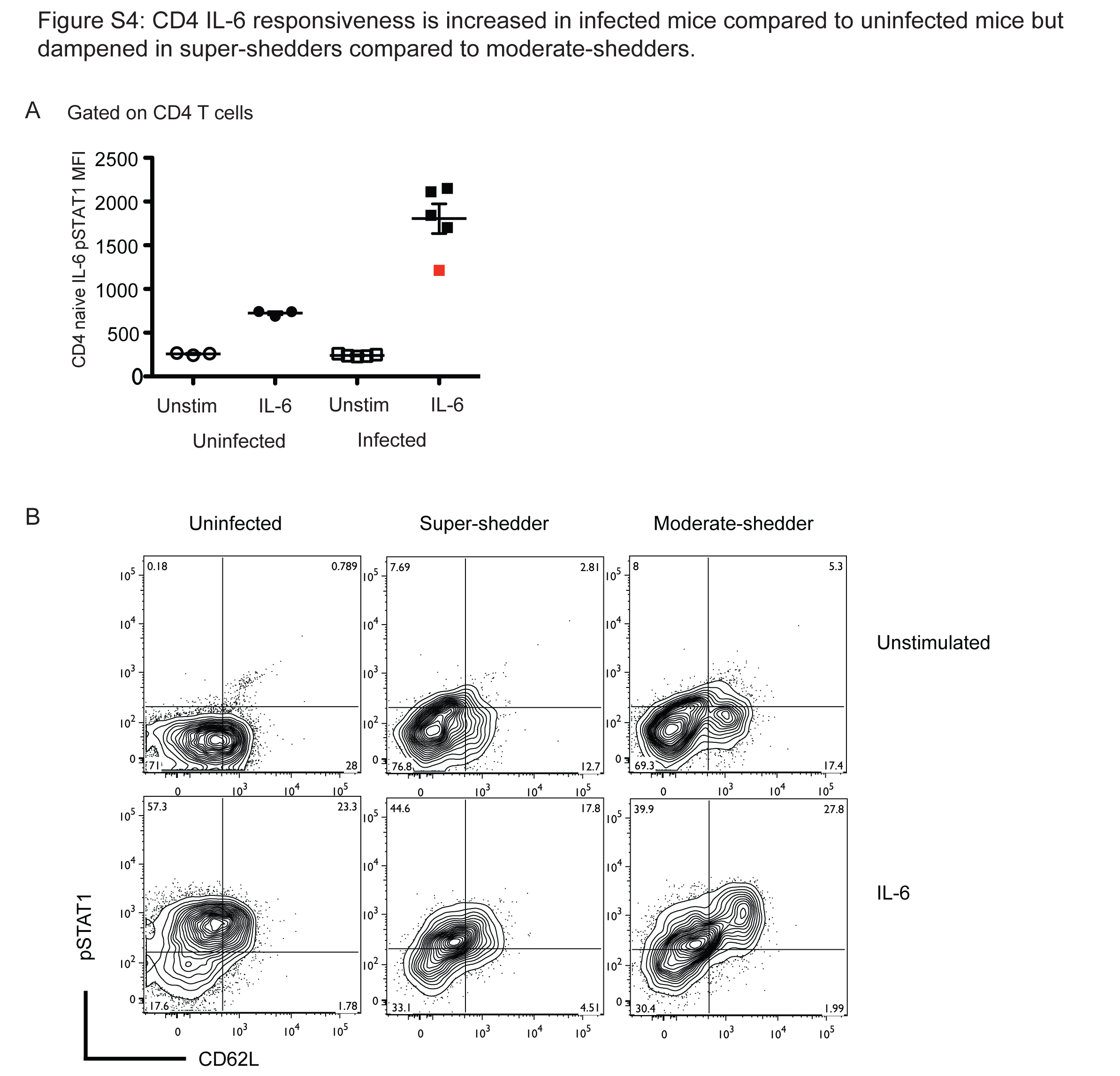

Supplement: Figure S4 — CD4 IL-6 responsiveness is increased in infected mice compared to uninfected mice but dampened in super-shedders compared to moderate-shedders. A, B: Splenocytes from 3 uninfected and 5 30-day infected mice were recovered and stimulated with 40 ng/mL IL-6 for 15 minutes then fixed and permeabilized. Data is representative of 3 independent experiments conducted with a total of 30 mice. A: pSTAT1 MFI of stimulated and unstimulated samples is shown. The red dot indicates the single super-shedder in the group of infected mice. B: Representative FACS plots of unstimulated and IL-6 stimulated samples from uninfected, super-shedder and moderate-shedder mice are depicted. X axis depicts CD62 Ligand expression and pSTAT1 MFI is shown on the Y axis. (TIF) [file ppat.1003408.s004.tif]

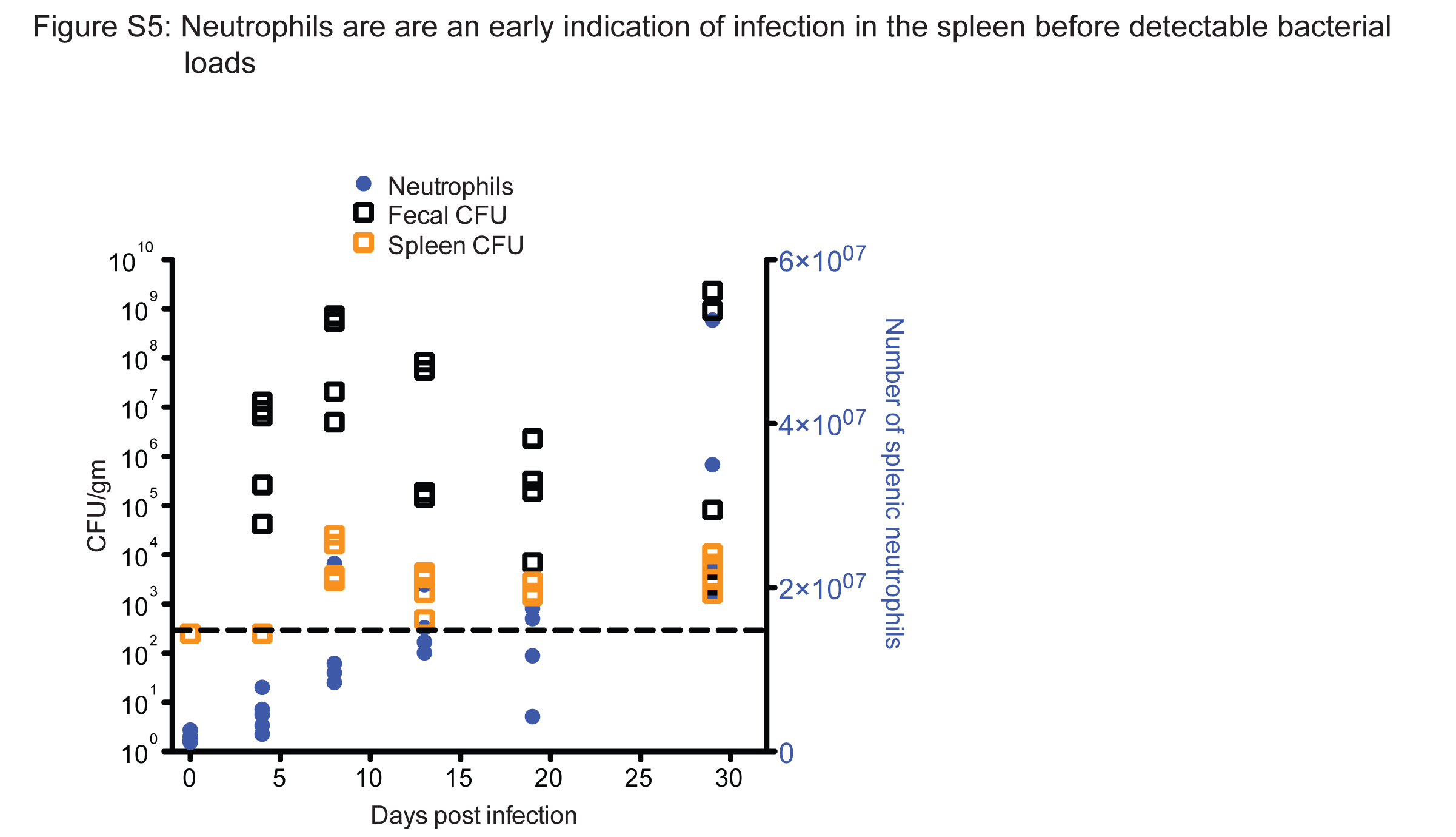

Supplement: Figure S5 — Neutrophils are an early indication of infection in the spleen, before detectable bacterial loads. A: Bacterial burden was quantified from the spleen and feces at days indicated post-infection. On the right y-axis, the number of Gr1+ cells (neutrophils) from the spleen were measured at the indicated time points. On the left y-axis bacterial load is represented on a log scale. Black squares represent fecal bacterial burden and yellow squares represent splenic bacterial burden at the indicated time points. (TIF) [file ppat.1003408.s005.tif]

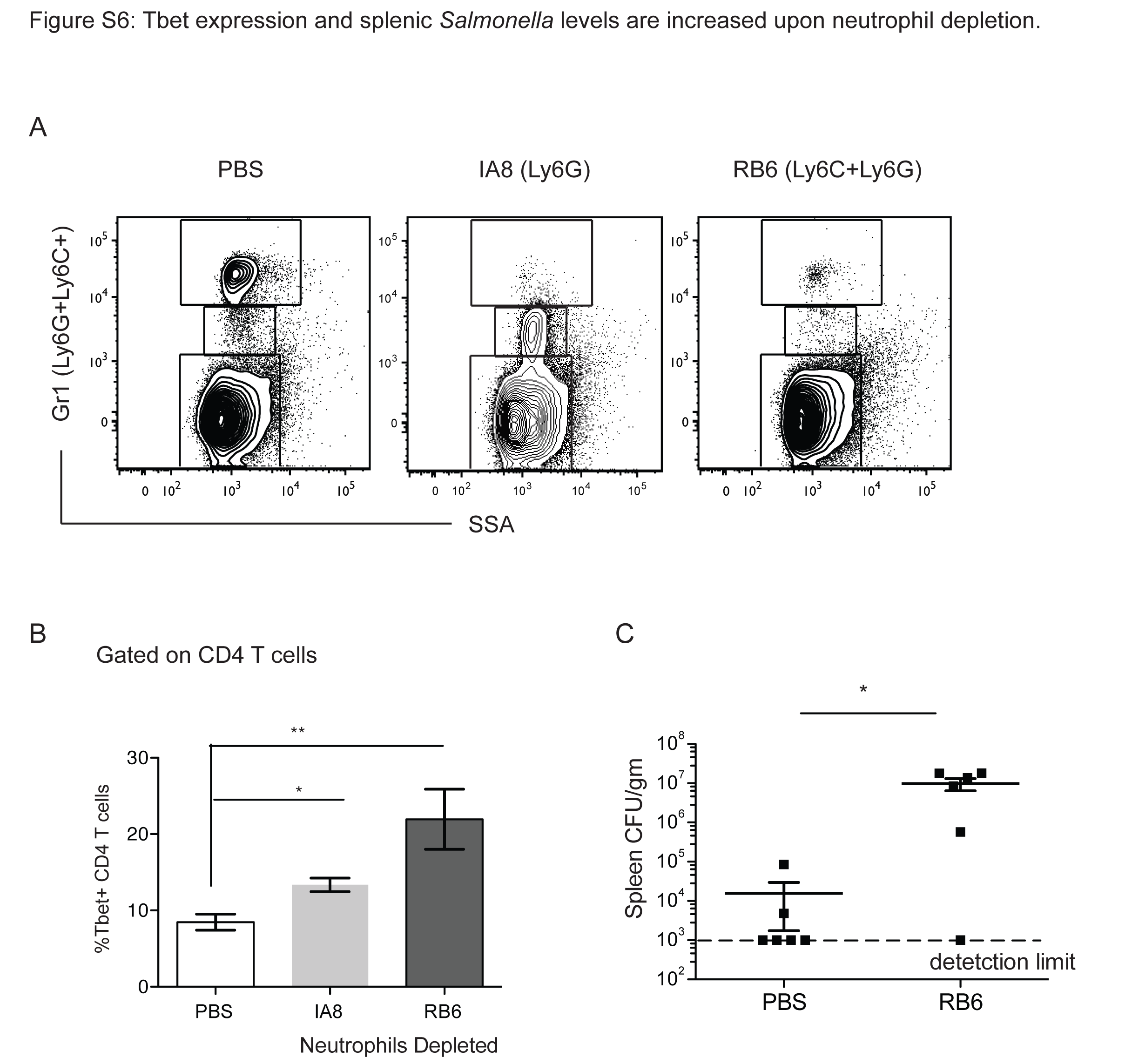

Supplement: Figure S6 — Tbet expression and splenic Salmonella levels are increased upon neutrophil depletion. Persistently infected mice were injected with neutrophil depletion antibodies (RB6 or IA8) or PBS controls for three days and sacrificed on the fourth. A. Representative FACs plots of splenocytes from mice treated with two different neutrophil depletion antibodies, IA8 which targets LY6G and RB6 which targets both Ly6C and Ly6G and PBS treated controls. B,C: Data is shown from 4–6 mice per condition and the experiment was repeated twice for a total of 10–12 mice per condition. Asterisks indicate p<0.05 calculated using two-tailed Mann-Whitney U test. B. TH1 cells are quantified as percentage of Tbet+ CD4 T cells of total CD4 T cells in the spleen. C. Splenic bacterial burden was measured in RB6 treated and PBS control mice. (TIF) [file ppat.1003408.s006.tif]

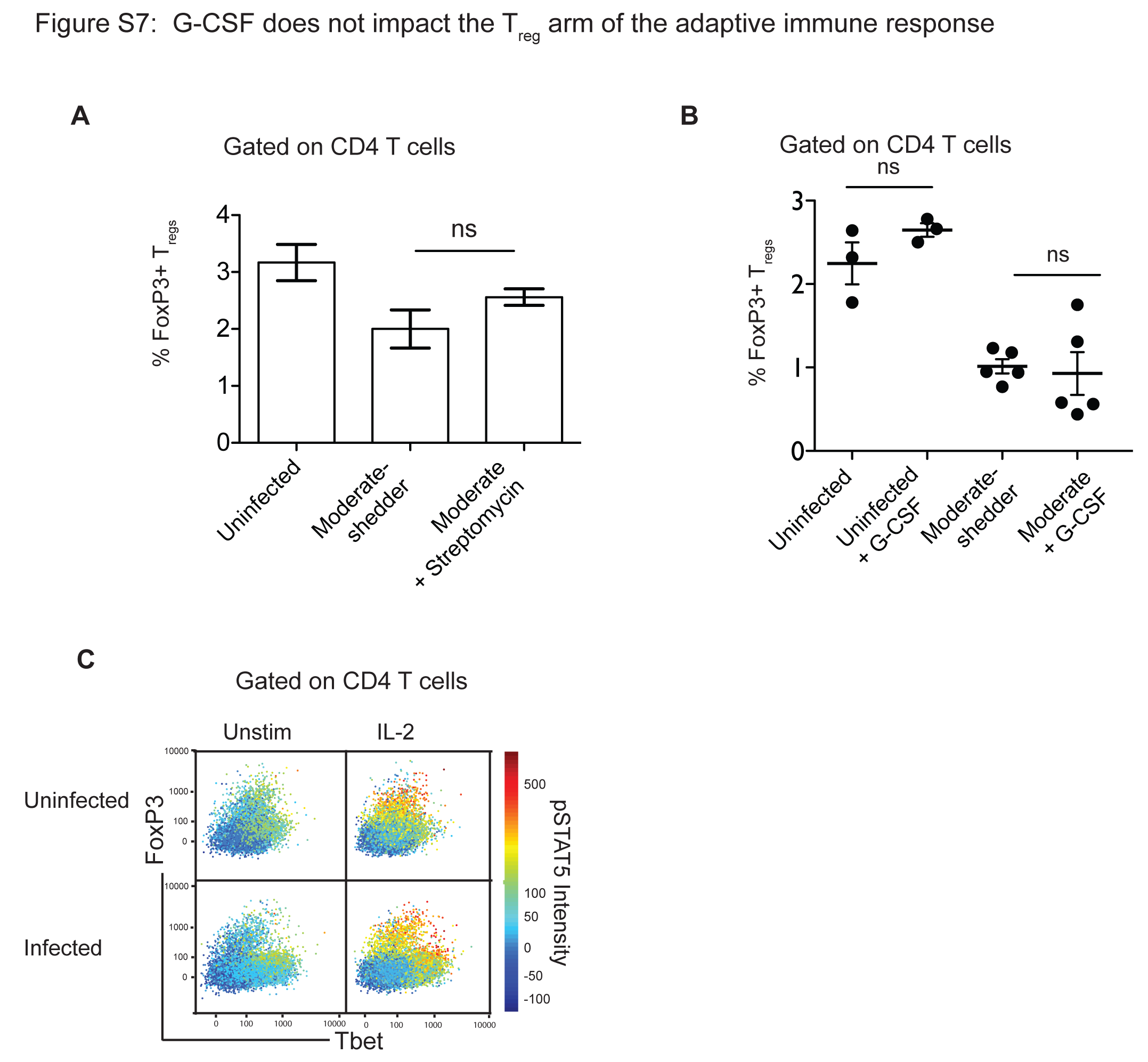

Supplement: Figure S7 — G-CSF does not impact the Treg arm of the adaptive immune response. A: Splenocytes from uninfected, moderate-shedder and streptomycin-treated moderate-shedder mice were collected as detailed in Figure 3 and Tregs were quantified as a percentage of total CD4 T cells. Data is shown from 3–5 mice per condition and the experiment was repeated twice for a total of 8–10 mice per condition. B. Uninfected and 30 day infected moderate-shedder mice were injected with G-CSF for 3 days and sacrificed as described in Figure 5. Tregs were quantified as a percentage of CD4 T cells in the spleen. Data shown is representative of 2 independent experiments with a total of 8–10 mice in each condition. C: Single cell suspensions were collected from the mesenteric lymph nodes of infected mice and stimulated ex vivo for 15 mins with 40 ng/ml IL-2, then fixed and permeabilized. Warmer colours indicate higher pSTAT5 MFI. Data shown is from a single mouse in stimulated and unstimulated conditions. It is representative of many independent experiments with a total of 30 mice. (TIF) [file ppat.1003408.s007.tif]

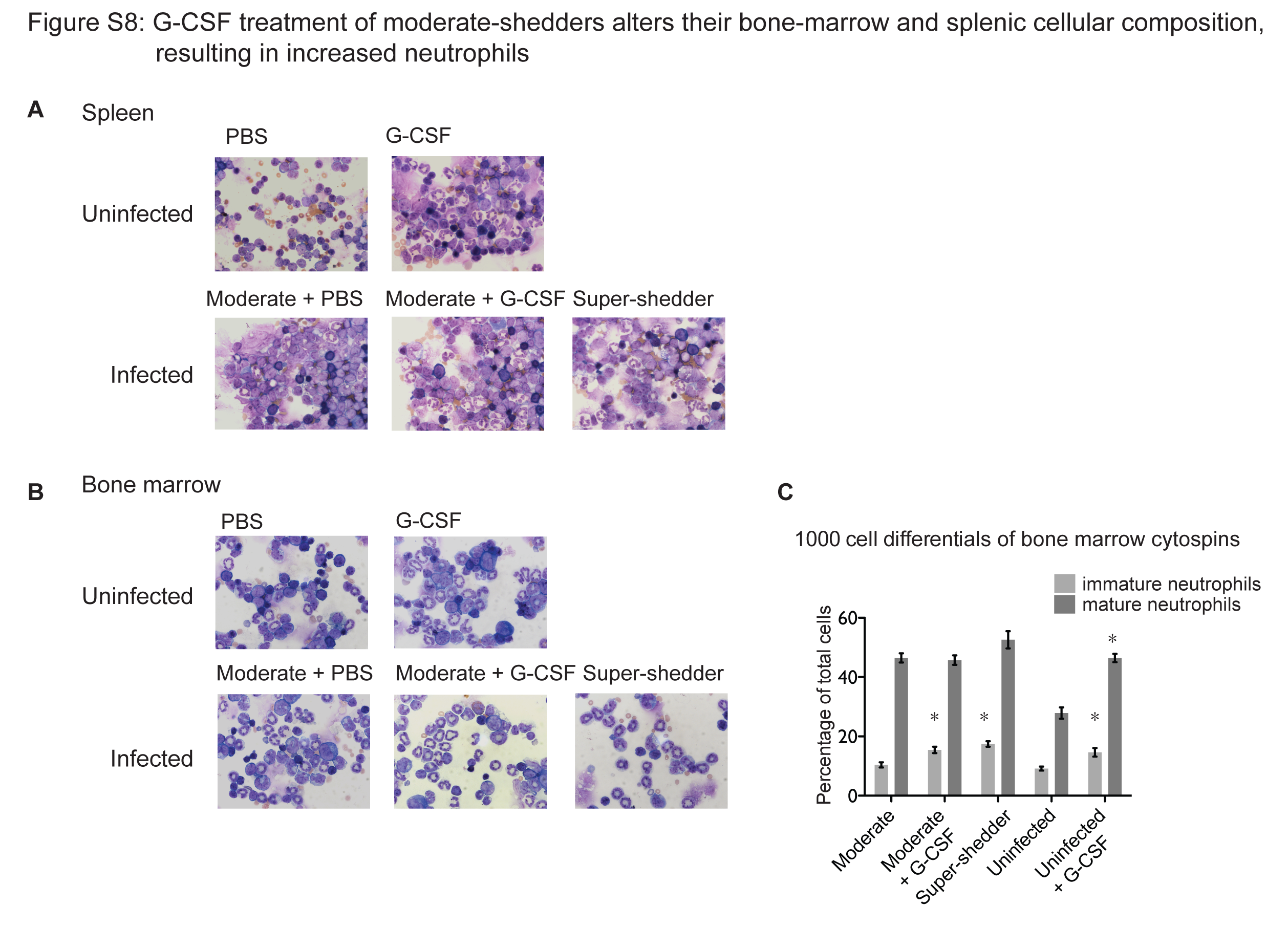

Supplement: Figure S8 — G-CSF treatment of moderate-shedders alters their bone-marrow and splenic cellular composition, resulting in increased neutrophils. A,B: Cytospins were performed on spleen (A) and bone marrow (B) samples from uninfected and infected mice with G-CSF and control (PBS) injected mice and quantified using 1000 cell differentials. Images from representative samples are shown. C: Mature and immature neutrophils were quantified as a percentage of the total cells. Asterisks indicate p<0.05 calculated using two-tailed Mann-Whitney U test and comparing to moderate-shedder or uninfected samples. (TIF) [file ppat.1003408.s008.tif]

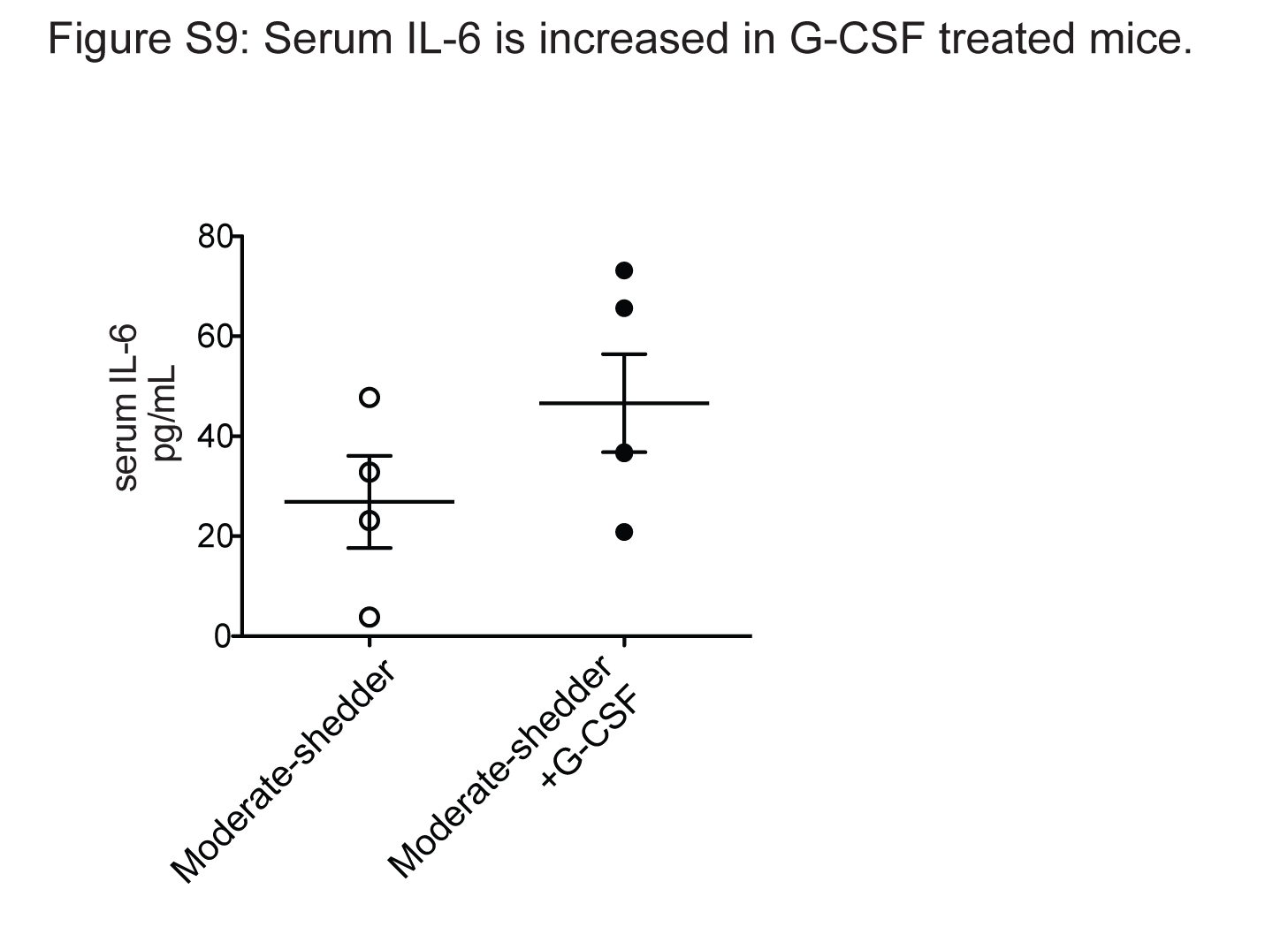

Supplement: Figure S9 — Serum IL-6 is increased in G-CSF treated mice. Serum IL-6 was quantified in G-CSF treated moderate shedders and control (PBS) moderate shedders. There were four mice in each condition and a one-tailed Mann-Whitney U test yielded a p value of 0.1. (TIF) [file ppat.1003408.s009.tif]

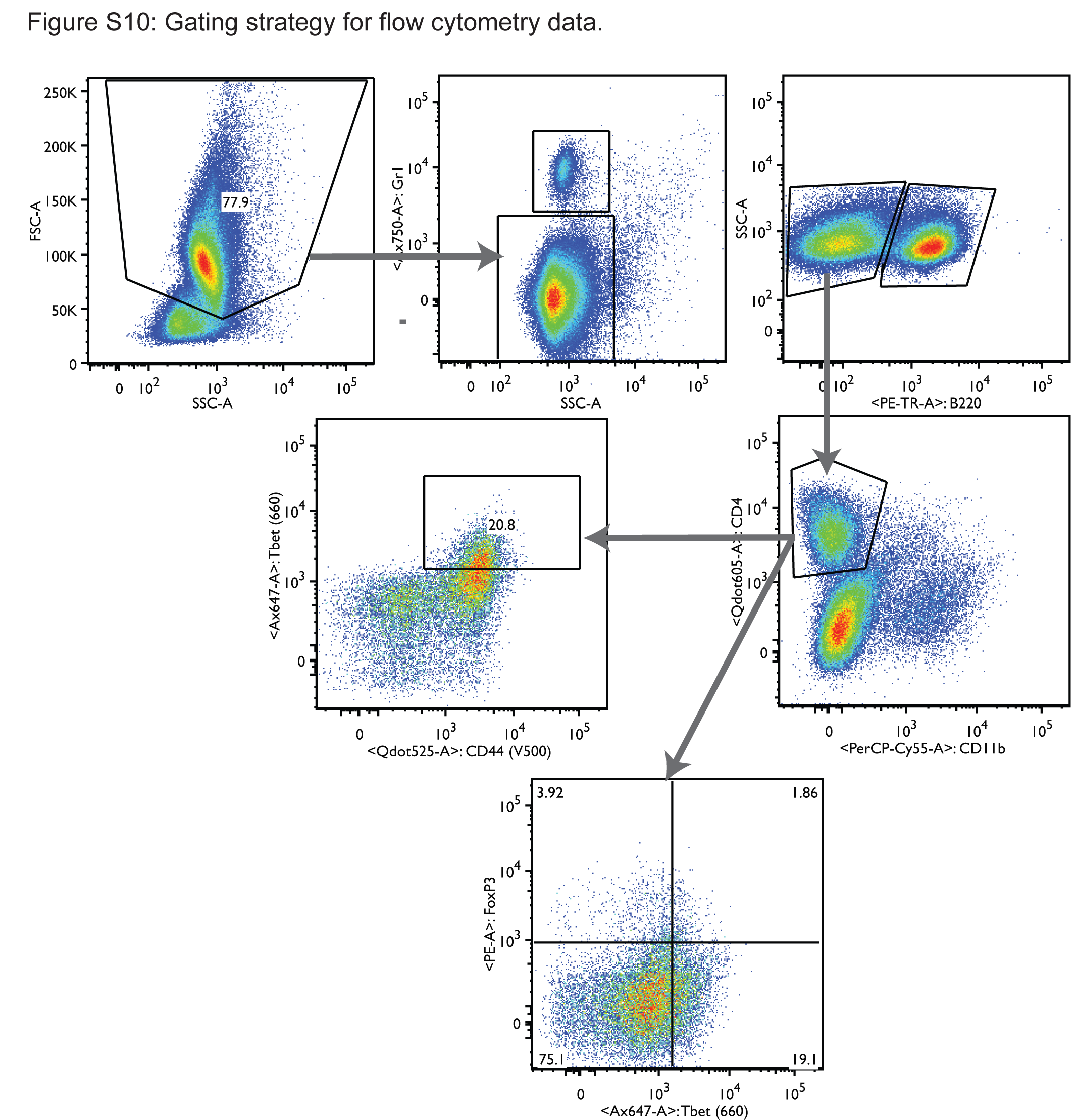

Supplement: Figure S10 — Gating strategy for flow cytometry data. Single cell suspensions were stained with the antibodies as mentioned in the materials and methods. Cells were gated on Forward and Side Scatter to exclude debris and red blood cells. After that, a hierarchical gating cluster was employed to identify Neutrophils, B cells, CD4 T cells and TH1 and Tregs as depicted. (TIF) [file ppat.1003408.s010.tif]
